# R‐Ras1 and R‐Ras2 regulate mature oligodendrocyte subpopulations

**DOI:** 10.1002/glia.24643

**Published:** 2024-11-19

**Authors:** Berta Alcover‐Sanchez, Gonzalo Garcia‐Martin, Víctor Paleo‐García, Ana Quintas, Ana Dopazo, Agnès Gruart, José María Delgado‐García, Pedro de la Villa, Francisco Wandosell, Marta P. Pereira, Beatriz Cubelos

**Affiliations:** ^1^ Centro de Biologia Molecular Severo Ochoa (CBM) CSIC Universidad Autonoma de Madrid Madrid Spain; ^2^ Departamento de Biología de Sistemas Universidad de Alcalá Madrid Spain; ^3^ Grupo de Neurofisiología Visual Instituto Ramón y Cajal de Investigación Sanitaria (IRYCIS) Madrid Spain; ^4^ Genomics Unit Centro Nacional de Investigaciones Cardiovasculares Carlos III (CNIC) Madrid Spain; ^5^ Division of Neurosciences Pablo de Olavide University Seville Spain; ^6^ Alzheimer's Disease and Other Degenerative Dementias Centro de Investigación Biomédica en Red de Enfermedades Neurodegenerativas (CIBERNED) Madrid Spain; ^7^ Instituto Universitario de Biología Molecular Universidad Autónoma de Madrid Madrid Spain

**Keywords:** mature oligodendrocytes, myelin, R‐Ras, specification, subpopulation

## Abstract

In the mammalian central nervous system, axonal myelination, executed by mature oligodendrocytes (MOLs), enables rapid neural transmission. Conversely, myelin deficiencies are hallmark features of multiple sclerosis, optic neuromyelitis, and some leukodystrophies. Recent studies have highlighted that MOLs are heterogeneous; however, how MOL subpopulations are specified and balanced in physiological settings is poorly understood. Previous works have demonstrated an essential role of the small GTPases R‐Ras1 and R‐Ras2 in the survival and myelination of oligodendrocytes. In this study, we aimed to determine how R‐Ras1 and R‐Ras2 contribute to the heterogeneity of MOL subpopulations. Our results evidence that R‐Ras1 and R‐Ras2 affect specification into the distinct subpopulations MOL1, MOL2, and MOL5/6, which in turn vary in their dependence of these GTPases. In R‐Ras1 and/or R‐Ras2 mutant mice, we observed an increase in the MOL1 subpopulation and a decrease in the MOL2 and MOL5/6 subpopulations. We identified R‐Ras1 and R‐Ras2 as key elements in balancing the heterogeneity of MOLs. Our results contribute to the understanding of the molecular mechanisms underlying the heterogeneity of MOLs and the myelination processes, which is crucial for innovating regenerative therapies for nervous system disorders.

## Main Points

- R‐Ras1 and R‐Ras2 are differentially expressed in mature oligodendrocytes subpopulations MOL1, MOL2 and MOL5/6.
- Lack of R‐Ras1 and R‐Ras2 produces an imbalance in mature oligodendrocyte subpopulation proportions.

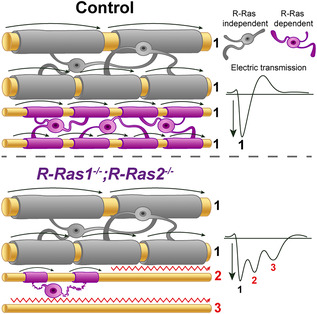

## 1 INTRODUCTION

Oligodendrocytes are specialized glial cells responsible for the myelination process in the central nervous system (CNS) of mammals (Nave & Werner, 2014). Oligodendrocytes insulate axons and help maintain their metabolism (Saab et al., 2013; Simons & Nave, 2016) by extending their membranous processes to wrap around axons and form myelin sheaths (Stadelmann et al., 2019). Myelin synchronizes electrical impulses between different regions in the CNS, integrating sensory, motor, and cognitive information. Across the CNS, axons vary in number, length, thickness, and distribution of myelin sheaths (Hughes et al., 2018; Tomassy et al., 2014). Thus, even subtle changes in axon myelination patterns can modify synchronization of nerve impulses (Fields, 2008).

During development, oligodendrocyte progenitor cells (OPCs) generated in the ganglionic eminences migrate throughout the CNS and give rise to mature oligodendrocytes (MOLs) that carry out critical physiological functions in different regions of the CNS (Chapman et al., 2013; Kessaris et al., 2006; Klämbt, 2009; Leong et al., 2014; Tekki‐Kessaris et al., 2001). During differentiation, oligodendrocytes gradually lose their proliferative and migratory capacity as they mature and undergo changes in morphological complexity and gene expression (Barateiro & Fernandes, 2014).

Del Río‐Hortega identified that oligodendrocyte populations myelinate axons of different diameters (Pérez‐Cerdá et al., 2015; Simons & Nave, 2016). In recent years, techniques such as single‐cell RNA sequencing have unraveled the molecular heterogeneity of oligodendrocytes (Bruggen et al., 2022; Hilscher et al., 2022; Seeker et al., 2023; Zeisel et al., 2015). Not only have such studies enabled the identification of subpopulations with distinct gene expression profiles, but also suggested specialized functional capabilities of oligodendrocyte beyond their traditional role in myelination (Marques et al., 2016). Recently, a high degree of functional specification in MOL populations has been suggested, reflected as associations with the local environment and regulatory interactions with different cell types (Jäkel et al., 2019; Meijer et al., 2022).

To date, six transcriptionally distinct subpopulations of MOLs have been described (MOL1–6), with the most distinct genetic markers found in MOL1, MOL2, and MOL5/6.

Gene expression patterns suggest that while MOL1 might be related to both metabolic support and axonal stress response, MOL2 might be involved in neuronal connectivity and synapse establishment, whereas MOL5/6 might participate in myelin sheath formation and adaptive myelination (Hilscher et al., 2022; Marques et al., 2016). However, these hypothetical functions are solely based on gene expression data and still require validation through additional functional studies.

Imbalances in MOL subpopulations have been described in patients with multiple sclerosis and in spinal cord injuries (Falcão et al., 2018; Floriddia et al., 2020; Jäkel et al., 2019). These imbalances in turn result in changes in the proportions, distribution, and genetic identity of MOL subpopulations in response to tissue damage. In particular, MOL2 and MOL5/6 are replaced by other subpopulations that express genes related to neuroprotection and immune response (Falcão et al., 2018). Specifically, in cases of tissue damage such as in multiple sclerosis disease or spinal cord injury models, the MOL1 subpopulation, which usually constitutes a minor percentage of the total MOLs in the adult CNS, expands (Falcão et al., 2018; Floriddia et al., 2020; Hilscher et al., 2022).

R‐Ras1 and R‐Ras2 are membrane‐anchored intracellular signal transducers belonging to the Ras GTPase superfamily. In previous work, our laboratory demonstrated in vivo that these R‐Ras family members are expressed in oligodendrocytes and that they are essential in CNS myelination (Sanz‐Rodriguez et al., 2018). R‐Ras members share strong homology with classical Ras (55%–60% amino acid identity), and this allows them to activate the same effector proteins (Colicelli, 2004; Fritsch et al., 2013). They are activated by a wide variety of extracellular stimuli and participate in multiple signaling pathways to regulate cellular processes, such as cell growth and division, differentiation, and survival (Wennerberg et al., 2005).

Myelin deficiency due to an absence of R‐Ras1 and/or R‐Ras2 causes metabolic and energetic alterations in axons, leading to axon degeneration and ultimately loss of motor and sensory function (Alcover‐Sanchez et al., 2021; Sanz‐Rodriguez et al., 2018). However, some oligodendrocyte populations are capable of correctly myelinating axons even in the absence of R‐Ras1 and/or R‐Ras2, indicating that these GTPases play differential roles in the survival and differentiation of distinct MOL subpopulations. Guided by this finding, we aimed to unravel which role could be playing R‐Ras1 and R‐Ras2 over MOL subpopulations to ensure proper myelination. In this study, we demonstrated that both R‐Ras1 and R‐Ras2 were expressed in the MOL1, MOL2, and MOL5/6 subpopulations analyzed. Loss of R‐Ras1 and R‐Ras2 produced an overall loss of MOLs and a shift in the distribution of MOL1, MOL2, and MOL5/6 subpopulations. Finally, MOL imbalances produced by the absence of R‐Ras1 and/or R‐Ras2 correlated with deficiencies on the axonal conduction velocities of mutant mice lacking these GTPases. Taken together, our results place R‐Ras1 and R‐Ras2 as key elements to ensure a correct balance in MOL subpopulations. We believe that unraveling the molecular mechanisms involved in MOL heterogeneity will provide tools for a deeper understanding of oligodendrocyte physiology, which may be applicable to many myelin‐related neurodegenerative diseases.

## 2 MATERIALS AND METHODS

### 2.1 Mice

Animal procedures were approved by the corresponding institutional ethical committee (Centro de Biología Molecular Severo Ochoa, CBM) and performed in accordance with Spanish and European guidelines. All efforts were made to minimize animal suffering. All animals received water and food ad libitum and were housed on a 12 h light/dark cycle under pathogen–free conditions in a humidity‐ and temperature‐controlled room. Experiments were performed in adult male and female, in *wild type* (control) and mutant *R‐Ras1* ^*−/−*^ (*R‐Ras1KO*), *R‐Ras2* ^*−/−*^ (*R‐Ras2KO*), and *R‐Ras1* ^*−/−*^;*R‐Ras2* ^*−/−*^ (double knockout; *DKO*) mice. *R‐Ras1KO* and *R‐Ras2KO* mice were kindly provided by Prof. B. Alarcón (CBMSO, Spain). *DKO* mice were generated by backcrossing individual lines of *R‐Ras1KO* and *R‐Ras2KO* mice. Generation of mouse lines used for this work was previously described (Sanz‐Rodriguez et al., 2018). Animals were maintained in a C57BL/6J background.

### 2.2 Transmission electron microscopy

Following the protocol described in (Sanz‐Rodriguez et al., 2018), we generated cross‐sections from optic nerves of adult (P90) control, *R‐Ras1KO*, *R‐Ras2KO*, and *DKO* mice and conducted ultrastructural analyses using a JEM‐1010 electron microscope. Six random micrographs per mouse were taken along the whole length of the optic nerve using a CMOS 4 K × 4 K TemCam‐F416. Images were captured at 12,000× magnification.

For axonal caliber analyses, we chose random micrographs from each genotype and quantified the area for each axon. We identified individual axons as a single‐membrane circle‐like structure containing mitochondria and microtubules and classified axons according to their caliber and myelination state. For the caliber, we calculated the mean size of the largest axons (1.19 μm^2^) and classified the remaining axons in two groups according to this measure (thin axons <.60 μm^2^ and thicker axons >.60 μm^2^). We analyzed a total of 131 axons in control mice, 145 axons in *R‐Ras1KO* mice, 81 axons in *R‐Ras2KO* mice, and 218 axons in *DKO* mice. For the number of myelin wraps around the axons, we analyzed a total of 20 axons in controls, 27 axons in *R‐Ras1KO* mice, 23 axons in *R‐Ras2KO* mice, and 14 axons in *DKO* mice and used 3 animals per genotype.

### 2.3 Percoll oligodendrocyte isolation

After dissection, brains from adult mice were dissected and kept in cold phosphate‐buffered saline (PBS). To ensure that all MOL subpopulations were included (Marques et al., 2016), we harvested 0.0 to −2.5 mm slices from Bregma with the help of a brain matrix stand (rodent brain matrix‐adult mouse; 30 g; coronal‐RBM‐2000C catalog: #69022; ASI Instruments, Inc., Warren, MI). Mutant and control mouse brain slices were chopped into small pieces with micro knives (Fine Science Tools, Foster City, CA; catalog: #10316–14), and then digested in 1 mL of papain (1 mg/mL; Worthington, Lakewood, NJ; catalog: #LK003178) containing DNase I (0.01 mg/mL; Worthington; catalog: #LK003172) for 10 min at 37 °C. Samples were then centrifuged for 5 min at 1000 rpm at room temperature (RT), the supernatant was discarded, and the papain reaction was stopped by dilution with 1 mL of ovomucoid (1 mg/mL; Worthington; catalog: #LS00308) in 1× Dulbecco's Modified Eagle Medium. The tissue was mechanically disaggregated with a P1000 pipette and then filtered through a 70‐μm cell strainer (Corning catalog #:352350) previously humidified with 1× PBS and later centrifuged for 5 min at 1000 rpm. The pellet was resuspended in a 15 mL tube with 2.5 mL of a 40% Percoll solution (Sigma‐Aldrich, Saint Louis, MO; catalog: #P1644) in PBS; subsequently, 2.5 mL of cold PBS were gently added on the tube wall (Robinson et al., 2014). After 25 min of 1800 rpm centrifugation at RT, the supernatant was discarded and the pellet, enriched in oligodendrocytes, was resuspended either in 0.5% bovine serum albumin (BSA; NZYTech, Lisboa, Portugal; catalog: #MB04602) in PBS for flow cytometry, or in lysis buffer for western blot.

### 2.4 Flow cytometry

To block non‐specific binding, Percoll oligodendrocytes were resuspended in PBS‐0.5% BSA and incubated for 30 min on ice. To distinguish dead cells before permeabilization and fixation, samples were incubated in darkness for 30 min at 4 °C with 1 μg/mL Ghost Dye 780 (Tonbo Biosciences catalog: #13‐0865‐T100) in PBS‐0.5% BSA. Next, samples were centrifuged 5 min at 1000 rpm and the supernatant was discarded. Each sample was resuspended in PBS‐0.5% BSA and distributed in control and sample tubes for specific staining. Extracellular staining was performed by the addition of specific extracellular antibodies (Table 1) and incubated 30 min at 4 °C in darkness. Later, samples were centrifuged 5 min at 1000 rpm and then resuspended in 100 μL of Fixation/Permeabilization solution (eBioscience™ Foxp3/Transcription Factor Staining Buffer Set, Invitrogen, Waltham, MA; catalog: #00–5523) and incubated 30 min at 4 °C in darkness for fixation and permeabilization. After incubation, samples were centrifuged 5 min at 1500 rpm, resuspended in 200 μL of Permeabilization Buffer 1× (eBioscience™ Foxp3/Transcription Factor Staining Buffer Set, Invitrogen; catalog# 00–5523) with 2% fetal bovine serum and incubated for 30 min at RT in darkness. Samples were centrifuged 5 min at 1500 rpm and incubated with specific intracellular antibodies (Table 1) overnight at 4 °C in darkness. Then, samples were washed and incubated with specific fluorescent‐tagged secondary antibodies (Table 2) for 30 min at 4 °C in darkness. When the staining contained more than 2 antibodies, primary antibodies were conjugated with conjugation kits (Table 3). Finally, fixed labeled cells were analyzed on a FACSCanto II flow cytometer (Becton Dickinson, Franklin Lakes, NJ), and data was analyzed with FlowJo software (Tree Star).

**TABLE 1:** Primary antibodies used for flow cytometry.

Target antigen	Species	Provider	Catalog number	Concentration
ApoE	Rabbit	Abcam	ab183597	1:200
			RRID: AB_3331650	
Egr2	Rabbit IgG	Abcam	ab245228	1:100
			RRID: AB_2934181	
FosB	Mouse IgG	Abcam	ab11959	1:200
			RRID: AB_298732	
Klk6	Goat Polyclonal IgG	Thermo Fisher Scientific	PA5‐47239	1:100
			RRID: AB_2606241	
Klk6	Rabbit polyclonal IgG	EpigenTek	A66674	1:100
			RRID: AB_2934182	
Mog	Mouse IgG	Merck Millipore	MAB5680	1:200
			RRID: AB_1587278	
Ptgds	Rabbit polyclonal	Thermo Fisher Scientific	PA5‐95977	1:100
			RRID: AB_2807779	
Rab37	Rabbit IgG	Abcam	ab233114	1:200
			RRID: AB_3331649	
R‐Ras1	Rabbit polyclonal	Abcam	ab154962	1:100
			RRID: AB_2894924	
R‐Ras2	Mouse IgG1	ExBio	11‐722‐C100	1:100
			RRID:	
			AB_3083593	

**TABLE 2:** Secondary antibodies.

Antibody	Species	Provider	Catalog number	Concentration
Alexa Plus 405 anti‐rabbit IgG	Donkey	Thermo Fisher Scientific	A‐48258	1:200
			RRID: AB_2890547	
Alexa 488 anti‐rabbit IgG	Donkey	Thermo Fisher Scientific	A‐21206	1:200
			RRID: AB_2535792	
PE anti‐mouse IgG	Rat	BioLegend	406608	1:200
			RRID: AB_10551439	
Alexa 647 anti‐mouse IgG	Donkey	Thermo Fisher Scientific	A‐31571	1:200
			RRID: AB_162542	

**TABLE 3:** Conjugation kits.

Conjugation kit	Provider	Catalog number
Alexa Fluor® 488 Conjugation Kit (Fast) – Lightning‐Link®	Abcam	ab236553
PE / R‐Phycoerythrin Conjugation Kit – Lightning‐Link®	Abcam	ab102918
Alexa Fluor® 647 Conjugation Kit (Fast) – Lightning‐Link®	Abcam	ab269823
PE/Cy7® Conjugation Kit – Lightning‐Link®	Abcam	ab102903

### 2.5 Gating strategy

The following gating strategy was used to define each MOL subpopulation. First, cell population was selected based on its size and complexity. Subsequently, doublets and dead cells were discarded. Next, the Mog^+^ population was selected and, finally, populations positive for each MOL marker and/or R‐Ras1 and R‐Ras2 were identified. Positive selection of each subpopulation was determined relative to its corresponding negative control, which accounted for non‐specific signals generated by the secondary antibodies. For all markers analyzed, populations displaying signal intensities exceeding those of the negative control were classified as positive.

### 2.6 Western blot

Optic nerve and Percoll oligodendrocytes from adult mice (P90) were resuspended in lysis buffer (50 mM Tris pH 8.0, 150 mM sodium chloride, 1% NP40, 2 mM ethylenediaminetetraacetic acid, 0.1% sodium dodecyl sulfate, 0.5% deoxycholate, and protease inhibitor mixture; Roche catalog: #11697498001) and phenylmethanesulfonyl fluoride. Lysates were then denatured by boiling for 5 min in protein loading buffer (50 mM Tris–HCl pH 6.8, 2% sodium dodecyl sulfate, 10% glycerol, 1% β‐mercaptoethanol, 12.5 mM ethylenediaminetetraacetic acid, and 0.02% bromophenol blue) and resolved on 10%–12% sodium dodecyl sulfate–polyacrylamide gel electrophoresis. Gels were run at constant current starting at 90 or 100 V. After electrophoresis, samples were transferred by electroblotting onto a polyvinylidene difluoride membrane in a semi‐dry electroblotting system (Trans‐Blot Turbo; Bio‐Rad Laboratories, Hercules, CA) at 1.2 mA/cm^2^ for 35–40 min. Nonspecific protein binding was blocked by incubating the membrane with 5% non‐fat milk in 0.1% TBS‐Tween‐20 for 1 h at RT. Membranes were incubated overnight with the pertinent primary antibodies in blocking buffer (Table 4). After washing, blots were incubated for 1 h with corresponding peroxidase‐conjugated secondary antibodies (Table 5). Labeled proteins were detected with the Chemiluminescence Reagent ECL (GE HealthCare Technologies Inc., Chicago, IL).

**TABLE 4:** Primary antibodies used for western blot.

Target antigen	Species	Provider	Catalog number	Concentration
ApoE	Rabbit	Abcam	ab183597	1:200
			RRID: AB_3331650	
Egr2	Rabbit IgG	Abcam	ab245228	1:500
			RRID: AB_2934181	
Gapdh	Mouse IgG1	Santa Cruz Biotechnology	sc‐365062	1:1000
			RRID: AB_10847862	
Fosb	Mouse IgG	Abcam	ab11959	1:200
			RRID: AB_298732	
Klk6	Rabbit IgG	EpigenTek	A66674	1:1000
			RRID: AB_2934182	
Mog	Mouse IgG	Merck Millipore	MAB5680	1:5000
			RRID: AB_1587278	
Ptgds	Rabbit polyclonal	Invitrogen, Thermo Fisher Scientific	PA5‐95977	1:1000
			RRID: AB_2807779	
Rab37	Rabbit IgG	Abcam	ab233114	1:200
			RRID: AB_3331649	

**TABLE 5:** Secondary antibodies.

Antibody	Species	Provider	Catalog number	Concentration
HRP‐anti‐mouse	Goat	Santa Cruz Biotechnology	Sc‐2005	1:5000
			RRID: AB_631736	
HRP‐anti‐rabbit	Goat	SouthernBiotech	4030‐05	1:5000
			RRID: AB_2687483	

### 2.7 Quantitative reverse transcription polymerase chain reaction (RT‐qPCR)

Complementary DNA (cDNA) was synthesized from total RNA from oligodendrocytes enriched by Percoll gradients or obtained by in vitro cultures. This was done using the SuperScript™ IV VILO™ Master Mix + ezDNAse (Thermo Fisher Scientific, catalog: #11766050) in a final reaction volume of 20 μL, processed in a thermal cycler (Applied Biosystems™ 2700). For qPCR expression profiling, the SsoFast™ EvaGreen® Supermix reagent (Bio‐Rad; catalog: #1725201) was used, and reactions were conducted on a CFX Opus 384 Real‐Time PCR System (Bio‐Rad) in 384‐well plates (Bio‐Rad; catalog: #HSP3905). Each qPCR reaction contained 5 ng of cDNA in a final volume of 10 μL. To control for genomic DNA contamination, the ValidPrime assay was performed (Laurell et al., 2012). Relative quantification of gene expression was carried out using the 2^−ΔΔCq^ method (Livak & Schmittgen, 2001), with *Gapdh* used as the reference gene for sample normalization. Gene expression levels in the control groups were normalized to 1. The same cDNA samples used for qPCR profiling were also analyzed by end‐point RT‐PCR. The RT‐qPCR primers used in this study were as follows (5' to 3'): *Egr2* (FW: GACCCCTGGATCTCCCGTAT, RV: AGGGTACTGTGGGTCAATGG), *Klk6* (FW: TGGGGAAACACAACCTACGG, RV: GTTGTAGCGGGGATGGACAA), *Ptgds* (FW: TCAGTGGTGGAGGCCAACTA, RV: GGTTCTGCTGTAGAGGGTGG), *Fosb* (FW: CCGAGAAGAGACACTTACCCC, RV: ACGGTTCCTGCACTTAGCTG), *Cdkn1c* (FW: CAGGGTGTCCCTCTCCAAAC, RV: GCCGTTAGCCTCTAAACTAACTC), and *Apoe* (FW: CACATTGCTGACAGGATGCCTA, RV: TCCCAGGGTTGGTTGCTTTG).

### 2.8 Immunohistochemistry

Animals were anesthetized (ketamine/xylazine) and perfused transcardially with 0.1 M PBS (pH 7.4) followed by PBS‐4% paraformaldehyde. Perfused tissues were removed and postfixed in PBS‐4% paraformaldehyde at 4 °C overnight, then cryoprotected in 30% sucrose in PBS and embedded and frozen in Optical Cutting Temperature solution (Tissue‐Tek; catalog: #4583). Next, tissues were sectioned on a cryostat to produce 25 μm cryosections on Superfrost Plus microscope slides (Thermo Fisher Scientific). Sections were blocked for 1 h at RT with 10% fetal bovine serum in PBS‐0.5% Triton‐X 100 (blocking solution) and then incubated overnight at 4 °C with primary antibodies (Table 6). After 3 washes, fluorescent‐tagged secondary antibodies (Table 7) were applied for 3 h in darkness at RT, and sections were counterstained with DAPI (Sigma‐Aldrich; catalog: #32670) and mounted in Aqua‐polymount mounting medium (PolyScience, Niles, IL; catalog: #18606).

**TABLE 6:** Primary antibodies used for immunohistochemistry.

Target antigen	Species	Provider	Catalog number	Concentration
Egr2	Rabbit IgG	Abcam	ab245228	1:200
			RRID: AB_2934181	
Klk6	Goat Polyclonal IgG	Thermo Fisher Scientific	PA5‐47239	1:100
			RRID: AB_2606241	
Klk6	Rabbit IgG	EpigenTek	A66674	1:200
			RRID: AB_2934182	
Olig2	Mouse IgG	Merck Millipore	MABN50	1:500
			RRID: AB_10807410	
Ptgds	Rabbit polyclonal	Invitrogen, Thermo Fisher Scientific	PA5‐95977 RRID: AB_2807779	1:200

**TABLE 7:** Secondary antibodies.

Antibody	Species	Provider	Catalog number	Concentration
Alexa 488 anti‐rabbit IgG	Donkey	Thermo Fisher Scientific	A‐21206 RRID: AB_2535792	1:500
Alexa 594 anti‐mouse IgG	Donkey	Thermo Fisher Scientific	A‐21203 RRID: AB_141633	1:500

### 2.9 Confocal microscopy

Fluorescence images from brain sections were obtained using either a confocal multispectral Leica TCS SP5 system (Leica Microsystems, Wetzlar, Germany) or a Stellaris 8 system (Leica Microsystems) controlled by LAS X 2.7 software (Leica Microsystems). Image acquisition was performed sequentially using a 20×/0.7NA dry objective or 40×/1.3NA oil objective and appropriate fluorochrome excitation lines (405, 488, and 594 nm for DAPI, Alexa‐488, and Alexa‐594, respectively).

### 2.10 Oligodendrocyte culture in vitro

#### 2.10.1 Primary cell cultures

OPCs were isolated from the cerebral cortex of control P2 mice pups, following an adapted protocol for OPC isolation (McCarthy and de Vellis, 1980; Molina‐Holgado et al., 2002; Medina‐Rodriguez et al., 2013; Murcia‐Belmonte et al., 2014). Purified OPCs were seeded into 6‐well plates previously buffered with 10 μg/mL poly‐L‐lysine (Sigma‐Aldrich; catalog: #P2636), 10 μg/mL polyornithine (Sigma‐Aldrich; catalog: #P3655) and 10 μg/mL laminin (Sigma‐Aldrich; catalog: #L2020) in OPC medium (Dulbecco's Modified Eagle Medium 10% fetal bovine serum, 2 mM glutamine, 0.6% glucose, and antibiotics [0.01% streptomycin and 100 U/mL penicillin G]). The next day, cell wells were transfected with plasmids encoding GFP (control) or a combination of GFP, R‐Ras1, and R‐Ras2 (Addgene, Watertown, MA, USA; GFP: #11153; R‐Ras1: #14727; R‐Ras2: #102335). Transfection was performed using Lipofectamine 2000 (Thermo Fisher Scientific; catalog: #11668019) according to the manufacturer's recommendations. After 6 h in transfection medium, cells were recovered in OPCs medium. The next day, cells were passaged in oligodendrocyte differentiation medium (Basal Medium Eagle; [Thermo Fisher Scientific; catalog: #41010], Ham's F‐12 Nutrient Mix [Gibco; catalog: #21765], 100 μg/mL holotransferrin [Sigma‐Aldrich; catalog: #T0665], 20 μg/mL putrescine [Sigma‐Aldrich; catalog: #P5780], 60 ng/mL progesterone [Sigma‐Aldrich; catalog: #P6149], 40 ng/mL sodium selenite [Sigma‐Aldrich; catalog: #S5261], 25 μg/mL insulin [Sigma‐Aldrich; catalog: #I1882], 800 ng/mL T4 [Sigma‐Aldrich; catalog: #T1775], 2 mM glutamine, 0.6% glucose, and antibiotics [streptomycin 0.01% and penicillin G 100 U/mL]). Cells were incubated at 37 °C in a 5% CO2 environment. Around DIV7, cells were either fixed for immunocytochemical staining or lysed for RNA extraction for RT‐qPCR experiments.

### 2.11 In vivo electrophysiology

#### 2.11.1 Visual evoked potentials (VEPs) of lateral geniculate nucleus (LGN)

In accordance with a previous study (Sanz‐Rodriguez et al., 2018), control (*n* = 9), *R‐Ras1KO* (*n* = 10), *R‐Ras2KO* (*n* = 12), and *DKO* (*n* = 12) mice were prepared for chronic recording of field potentials evoked at the LGN by visual stimulation (flashes of light). To this end, animals were anesthetized with 4% chloral hydrate and stereotaxically implanted with two recording electrodes in the dorsal part of the LGN 2.2–2.5 mm posterior to the Bregma, 2.0 mm lateral to the midline, and − 2.5 mm depth from the brain surface (Paxinos & Franklin, 2013). Electrodes were made from 50 μm Teflon‐coated tungsten wire (Advent Research Materials, Oxford, England). Two bare silver wires were affixed to the skull as ground. Electrodes were connected to a 4‐pin socket (RS‐Amidata, Madrid, Spain) that was later fixed with dental cement to the cranial bone. After surgery, animals were kept for 5 d in independent cages with ad libitum access to food and water for a proper recovery. Light stimulation was provided by a xenon arc lamp located 30 cm in front of the animals' eyes and lasted ~1 ms (photic stimulator; Cibertec, Madrid, Spain). For recordings, each alert behaving mouse was placed in a transparent box (5 × 5 × 5 cm), dark adapted for >30 min, and presented with a total of 20 stimuli at a rate of 3/min. This box was in the center of a larger container (30 × 30 × 30 cm) covered by polished aluminum walls. Photic stimulations were triggered from a programmable CS‐20 stimulator (Cibertec). Each animal received two stimulation sessions. We measured the conduction velocity of nerve impulses along the ON from the flash stimulation to the First Negative Component (tFNC), and then to the Late Negative Component (tLNC) in cases where successive negative waves were recorded.

#### 2.11.2 VEPs of visual cortex (VC)

Monocular VEPs were recorded in response to flashlight on the right eye. Chronic active electrode implantation was performed as described in previous studies (Milla‐Navarro et al., 2022). Control (*n* = 3) and *DKO* (*n* = 6) mice were anesthetized by intraperitoneal injection of 0.5 mL/150 g of saline solution (0.9% sodium chloride) containing ketamine/xylazine (100 mg/kg and 5 mg/kg, respectively; S. A. Barcelona, Spain). The hair was removed, and skin was cut at the top of the skull. A hole (1 mm in diameter) was stereotaxically drilled over the left primary VC (coordinates: −3.6 mm from Bregma and 2.4 mm lateral to left Lambda) due to the contralateral projection of the visual fields. Next, a sterilized stainless‐steel screw (1 mm diameter, 27 mm height; ZOEON, China) was implanted through the drilled hole and fixed with Duralay® dental self‐curing resin (Dental Mfg. Co. Worth, IL). The mice recovered from surgery for a week before VEP recording. To mitigate surgical pain, mice received oral meloxicam (Ceva, Barcelona, Spain) diluted (0.033 mg/mL) in drinking water for 3 d pre‐operation and 4 d post‐operation.

Mice were then kept in complete darkness for 10 min before signal recording. Recording of VEP was performed and signal acquisition, analysis, and storage were performed using Power‐Lab‐ADI® and Labchart® v8 software (ADInstruments, Oxford, UK). Recordings of the VEP signal underwent 10,000 times amplification, and corresponding high‐ and low‐pass filters were 5 and 500 Hz.

White light stimuli were derived from a homemade LED‐based Ganzfeld dome located 2 cm from the mouse eye. The flashlight intensity was calibrated with a calibrator (Gossen Mavo Monitor, Germany) based on ERG ISCEV standards adapted for mouse recordings. VC responses to 3 cd·s/m^2^ light intensity flashes applied every 5 s were recorded. At least 60 events were averaged. Latencies to the tFNC and tLNC components of the VEP were measured.

### 2.12 Statistical analysis

Quantitative data are shown as the mean ± standard deviation (SD). Differences between experimental groups were evaluated using a two‐tailed Student's *t*‐test or one‐way analysis of variance (ANOVA). Statistical numeric data are provided in the figure legends. (*) means *p* < .05; (**) means *p* < .01; and (***) means *p* < .001. Outliers were identified using boxplots. These outliers were discarded as being outside the median trend.

## 3 RESULTS

### 3.1 Absence of R‐Ras1 and/or R‐Ras2 has different effects on myelination of the optic nerve

**FIGURE 1:**
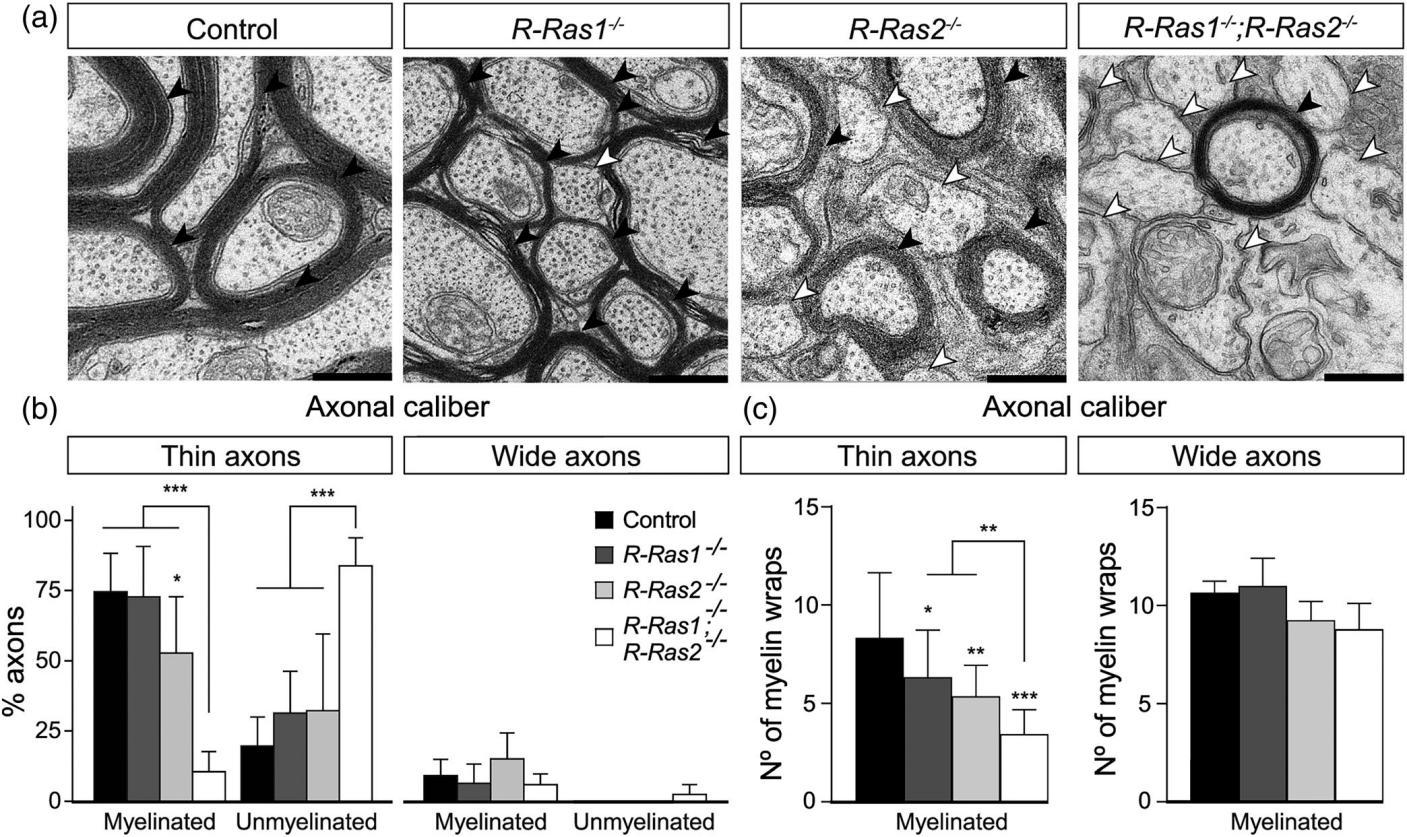
**The absence of R‐Ras1 and/or R‐Ras2 primarily affects the myelination of small‐caliber axons in the optic nerve.** (a) Optic nerve (ON) cross sections of adult (P90) control, *R‐Ras1* ^−/−^, *R‐Ras2* ^−/−^ and *R‐Ras1* ^−/−^; *R‐Ras2* ^−/−^ mice analyzed by transmission electron microscopy, showing a decrease in myelination of axons (white arrows). The presence of correctly myelinated axons was also observed (black arrows). (b) Classification of oligodendrocytes as thin and wide depending on the caliber of the axons they myelinate. The percentage of myelinated thin axons showed a reduction in *R‐Ras2* ^*−/−*^ mice relative to control mice (*p < .05) and on the *R‐Ras1* ^*−/−*^; *R‐Ras2* ^*−/−*^ mice relative to control and single mutant mice (***p < .001). The percentage of non‐myelinated thin axons showed a significant increase in *R‐Ras1* ^*−/−*^; *R‐Ras2* ^*−/−*^ mice relative to control and single mutant mice (***p < .001). No significant differences were found in the myelination of wide axons in any of the groups of mice analyzed. (c) Quantification of the number of myelin sheath wraps around the axon as a function of axonal caliber. Myelinated thin axons showed a significant reduction on the number of myelin wraps in *R‐Ras1* ^*−/−*^ mice relative to control (*p < .05), *R‐Ras2* ^*−/−*^ mice relative to control (**p < .01) and *R‐Ras1* ^*−/−*^; *R‐Ras2* ^*−/−*^ mice relative to control (***p < .001), *R‐Ras1* ^*−/−*^ (**p < .01) and *R‐Ras2* ^*−/−*^ (**p < .01) mice. No significant differences were found on the number of myelin wraps around wide axons in any of the groups of mice analyzed. Bar graphs represent mean ± SD. Scale bar, 500 nm. Two‐tailed Student's *t*‐test was used for statistical analysis. SD, standard deviation; *n* = 3 per genotype.

In previous work, we conducted transmission electron microscopy analysis to demonstrate that R‐Ras1 and R‐Ras2 are essential components of the myelination processes. Specifically, we observed differences in myelin ensheathment of retinal ganglion cell axons in adult *R‐Ras1KO*, *R‐Ras2KO*, and *DKO* mice relative to control mice (Alcover‐Sanchez et al., 2021; Sanz‐Rodriguez et al., 2018) (Figure 1a, white arrowheads). However, because correctly myelinated axons are present in the mutant mice, it is clear that the loss of R‐Ras1 and/or R‐Ras2 does not affect oligodendrocytes in the same way (Figure 1a, black arrowheads).

Following the classification system of del Río‐Hortega, we classified optic nerve oligodendrocytes as thin or wide based on the caliber of the myelinated axon. Overall, the percentage of myelinated thin axons was similar across control and *R‐Ras1KO* mice (74.50 ± 14% vs. 72.73 ± 18%) but significantly reduced in the *R‐Ras2KO* mice (52.79 ± 20%, *p* < .05). The proportion of myelinated thin axons in the *DKO* mice was also significantly smaller than that in control, *R‐Ras1KO*, and *R‐Ras2KO* mice (10.57 ± 7%, *p* < .001). Consequently, the percentage of un‐myelinated thin axons was significantly higher in the *DKO* mice compared with all other groups (83.77 ± 10% vs. 19.61 ± 10% for control mice, 31.33 ± 15% for the *R‐Ras1KO*, and 32.23 ± 28% for the *R‐Ras2KO* mice; *p* < .001). In contrast, the absence of R‐Ras1 and/or R‐Ras2 did not affect the myelination of thick axons (Figure 1b).

In parallel, we quantified the number of myelin sheath wrappings on both single mutant and *DKO* mouse axons. Relative to thin axons in control mice, mutant mice had significantly fewer myelin wraps (control: 8.35 ± 3.30; *R‐Ras1KO*: 6.35 ± 2.39 *p* < .05; *R‐Ras2KO*: 5.37 ± 1.57; *p* < .01; and *DKO* 3.44 ± 1.24; *p* < .001 relative to control and *p* < .01 relative to single mutant mice). Conversely, quantification of the number of myelin sheaths on the thicker axons did not reveal any significant differences between mutant and control mice (Figure 1c).

Taken together, these results suggest that the loss of R‐Ras1 and R‐Ras2 specifically affects the population of oligodendrocytes responsible for myelination of thin axons without affecting those responsible for myelination of thick axons. Based on these data, we hypothesize that these GTPases are differentially regulated in specific oligodendrocyte subpopulations.

### 3.2 Heterogeneous expression of R‐Ras1 and/or R‐Ras2 in mature oligodendrocyte populations

**FIGURE 2:**
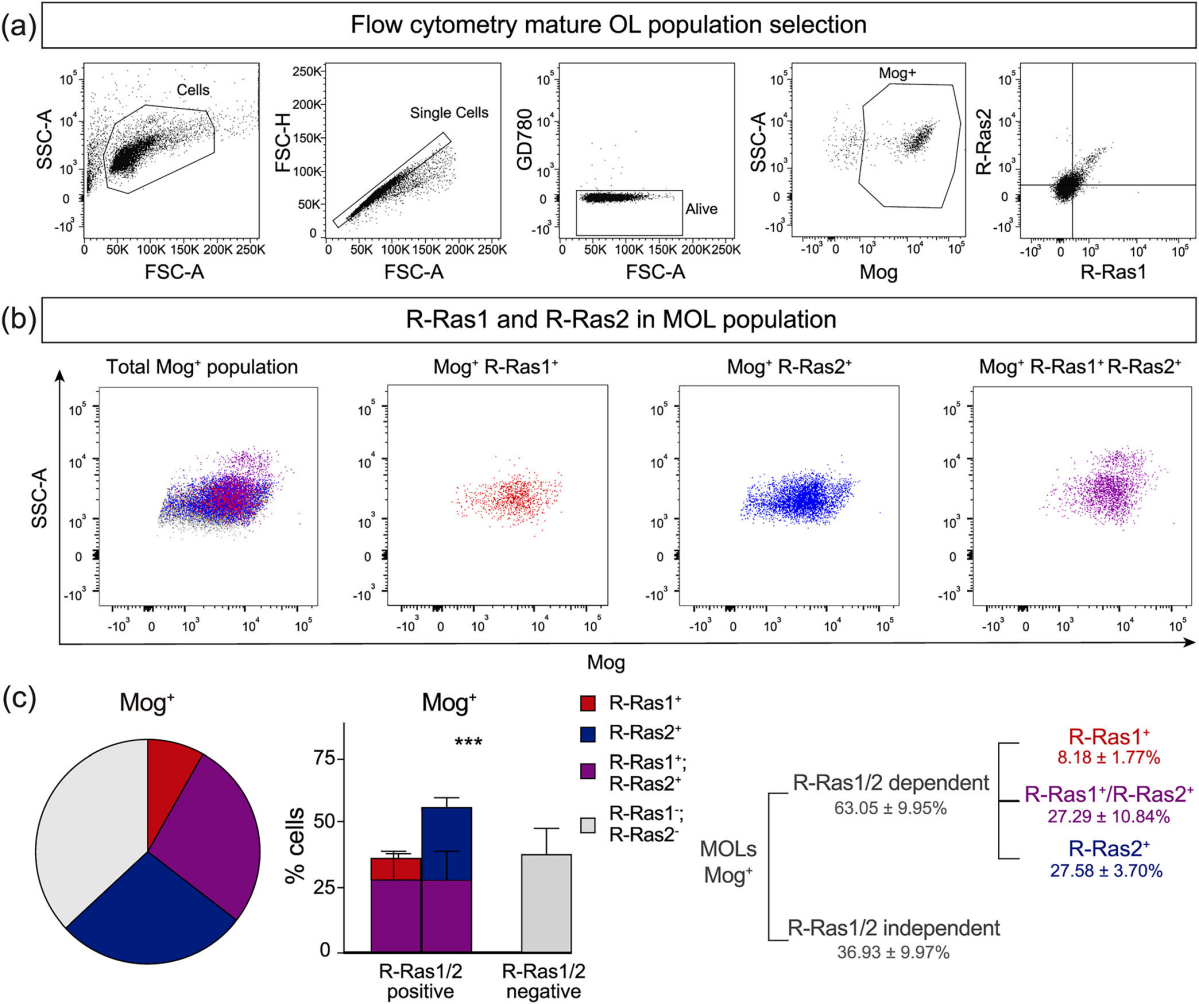
**R‐Ras1 and R‐Ras2 are differentially expressed in mature oligodendrocytes.** (a) Flow cytometry plots representative of the sequential selection of the mature oligodendrocyte (MOL) populations: Side‐scattered area (SSC‐A)/forward‐scattered area (FSC‐A) > forward‐scattered height (FSC‐H)/FSC‐A > Ghost dye 780 (GD780)/FSC‐A > SSC‐A/Mog > R‐Ras2/R‐Ras1. (b) Flow cytometry plots representative of the R‐Ras1 and/or R‐Ras2 subpopulations into which MOLs of enriched oligodendrocytes from adult (P90) mouse brains were classified. (c) Pie chart, bar graph and diagram showing differential expression of R‐Ras1 and/or R‐Ras2 in the MOL population (***p < .001). Bar graphs represent mean ± SD. One‐way analysis of variance (ANOVA) was used for statistical analysis. SD, standard deviation; *n* = 12 per genotype.

To assess R‐Ras1 and R‐Ras2 expression in the broader MOL population, we performed flow cytometry on oligodendrocytes enriched from the adult mouse brain as previously described (Marques et al., 2016) (See section Materials and Methods) and selected the MOL population using a sequential gating strategy based on the marker myelin oligodendrocyte glycoprotein (Mog) (Coffey & Mcdermott, 1997). Overall, R‐Ras1 and/or R‐Ras2 were expressed in roughly two thirds of Mog^+^ MOLs (63.05 ± 9.95%, Figure 2a), but with exclusive R‐Ras1 expression in 8.18 ± 1.77%, exclusive R‐Ras2 expression in 27.58 ± 3.70%, and co‐expression of R‐Ras1 and R‐Ras2 in 27.29 ± 10.84% of Mog^+^ MOLs (*p* < .001) (Figure 2b,c). These data show that variable expression of R‐Ras1 and R‐Ras2 demarcates distinct MOL subpopulations and also suggest that such variable expression confer unique properties to MOL subpopulations.

### 3.3 Heterogeneous expression of R‐Ras1 and/or R‐Ras2 in MOL1, MOL2, and MOL5/6 subpopulations

To investigate R‐Ras1 and R‐Ras2 expression in MOL1, MOL2, and MOL5/6 mouse brain subpopulations, we performed flow cytometry experiments by co‐staining MOL subpopulations with R‐Ras1 and R‐Ras2 subpopulation markers as previously described (Marques et al., 2016) (also see Materials and Methods). Specifically, we used the following enriched markers to detect MOL1: early growth response protein 2 (Egr2), MOL2 (Kallikrein‐6 [Klk6]), and MOL5/6 (prostaglandin D2 synthase [Ptgds]). (https://brainrnaseq.org/, http://linnarssonlab.org/oligodendrocytes/) (Floriddia et al., 2020) (Supplementary Figure 1).

**FIGURE 3:**
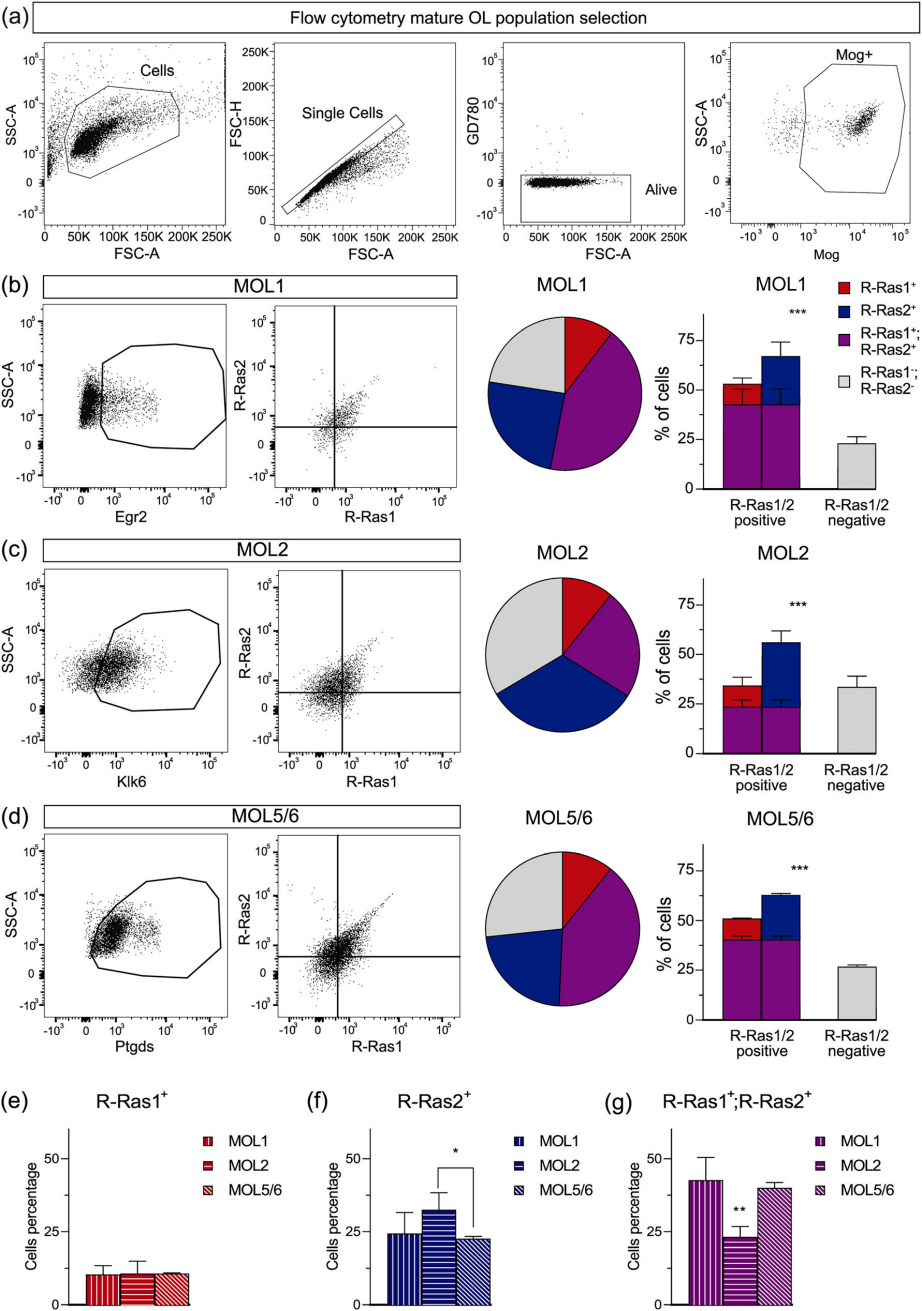
**MOL1, MOL2, and MOL5/6 subpopulations differentially express R‐Ras1 and/or R‐Ras2.** (a) Flow cytometry plots representative of the sequential selection of the mature oligodendrocyte (MOL) populations: Side‐scattered area (SSC‐A)/forward‐scattered area (FSC‐A) > forward‐scattered height (FSC‐H)/FSC‐A > Ghost dye 780 (GD780)/FSC‐A > SSC‐A/Mog. After selecting the global population of MOLs, positive subpopulation selection was performed. Representative flow cytometry plots of the (b) MOL1 subpopulation (Mog^+^Egr2^+^), (c) MOL2 subpopulation (Mog^+^Klk6^+^), and (d) MOL5/6 subpopulation (Mog^+^Ptgds^+^) with pie and bar graphs of the percentage of MOL cells expressing R‐Ras1 and/or R‐Ras2. R‐Ras1 and/or R‐Ras2 were expressed in different proportions in all the MOL subpopulations analyzed (***p < .001). (e) Bar graph showing the percentage of cells expressing R‐Ras1^+^ among the MOL1, MOL2 and MOL5/6 subpopulations. (f) Bar graph depicting the percentage of cells expressing R‐Ras2^+^ among the MOL1, MOL2 and MOL5/6 subpopulations, revealing an increase in the MOL2 subpopulation compared to MOL5/6 (*p < .05). (g) Bar graph illustrating the percentage of cells expressing R‐Ras1^+^ and R‐Ras2^+^ among the MOL1, MOL2 and MOL5/6 subpopulations, indicating a decrease in the MOL2 subpopulation compared to MOL1 and MOL5/6 (**p < .01). All bar graphs represent mean ± SD. For statistical analysis, a one‐way ANOVA was used for panels (b‐d), while a two‐tailed Student's *t*‐test was applied for panels (e‐g). SD, standard deviation; *n* = 3 adult (P90) mice.

First, these experiments showed that R‐Ras1 and/or R‐Ras2 are highly expressed in the MOL1, MOL2, and MOL5/6 subpopulations; however, we noticed variations across subpopulations (Figure 3a–c). Specifically, within the MOL1 subpopulation, ~10% of cells were exclusively R‐Ras1 positive, and ~ 24% exclusively R‐Ras2 positive, whereas ~43% of MOL1 cells were positive for both R‐Ras1 and R‐Ras2, and ~ 23% were negative for both (*p* < .001) (Figure 3a). In the MOL2 subpopulation, ~10% of cells were also exclusively R‐Ras1 positive, whereas ~33% were exclusively R‐Ras2 positive, 23% positive for both R‐Ras1 and R‐Ras2 and 33.53% negative for both (*p* < .001) (Figure 3b). In the MOL5/6 subpopulation, 10% of cells were again R‐Ras1 positive whereas 22% were exclusively R‐Ras2 positive, 40% positive for both R‐Ras1 and R‐Ras2 and 27% negative for both (*p* < .001) (Figure 3c).

Second, we compared the expression of R‐Ras1 and/or R‐Ras2 among the main MOL subpopulations. Our results indicated that the exclusive expression of R‐Ras1 did not significantly differ among the main subpopulations studied. Specifically, the percentage of R‐Ras1^+^ cells in MOL1 was 10.42 ± 3.01%, in MOL2 it was 10.72 ± 4.27%, and in MOL5/6 it was 10.73 ± 0.25% (Figure 3e).

In contrast, the exclusive expression of R‐Ras2 was elevated in the MOL2 subpopulation compared to MOL5/6. The percentage of R‐Ras2^+^ cells was 24.37 ± 7.19% in MOL1, 32.52 ± 5.85% in MOL2, and 22.60 ± 0.78% in MOL5/6 (*p* < .05) (Figure 3f).

The joint expression of R‐Ras1^+^ and R‐Ras2^+^ showed a significant reduction in the MOL2 subpopulation. Specifically, the percentage of cells jointly expressing R‐Ras1^+^ and R‐Ras2^+^ was 42.67 ± 7.80% in MOL1, 23.22 ± 3.56% in MOL2, and 40.03 ± 1.84% in MOL5/6 (*p* < .01) (Figure 3g).

### 3.4 MOL1, MOL2, and MOL5/6 subpopulations are affected in *R‐Ras1KO* and/or *R‐Ras2KO* mutant mice

**FIGURE 4:**
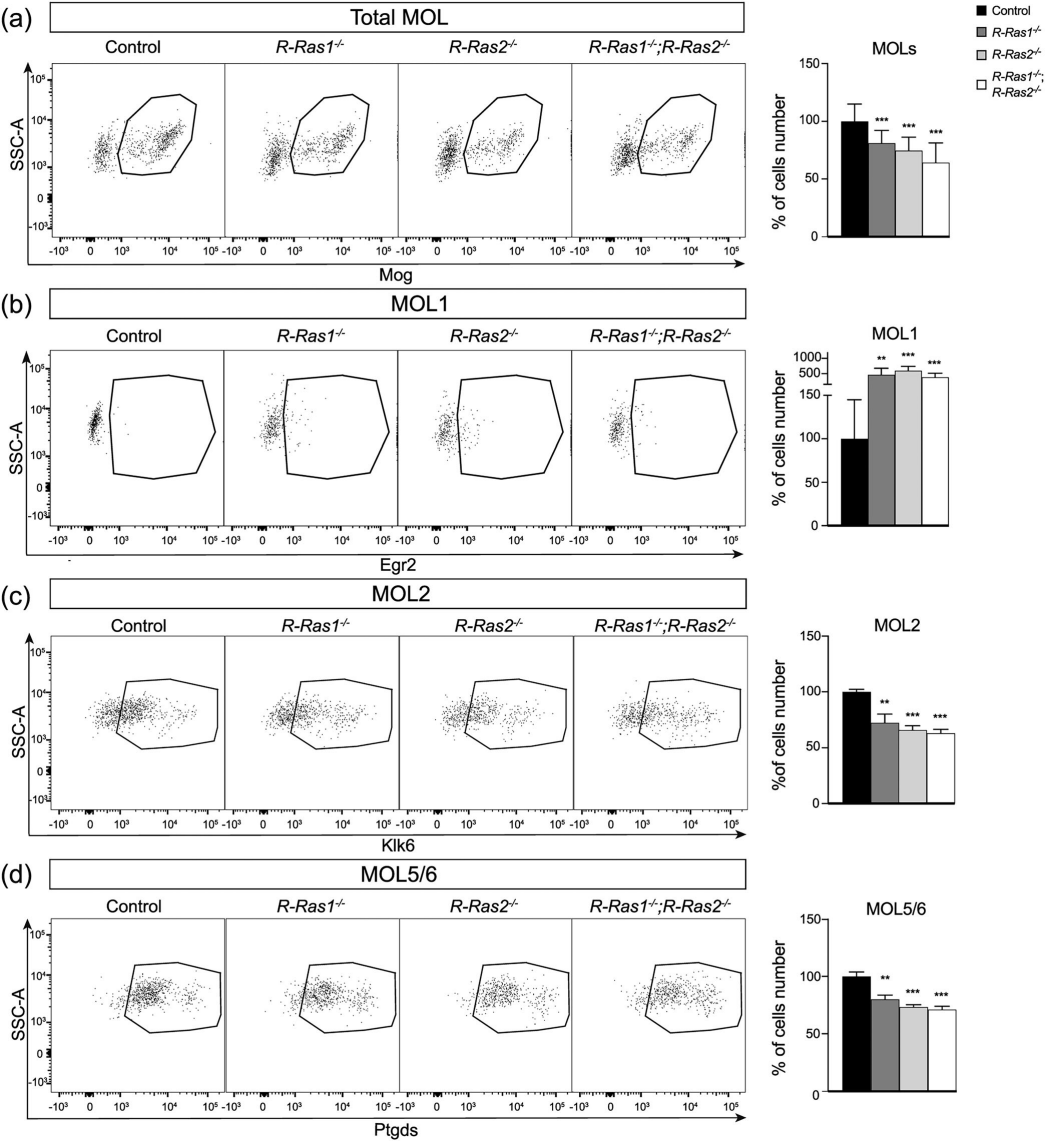
**Relative proportion of MOL1, MOL2 and MOL5/6 is altered in the absence of R‐Ras1 and/or R‐Ras2.** (a) Flow cytometry plots representative of total MOL population (Mog^+^) and bar graph showing MOL percentage in single and mutant adult mice relative to control. There was a significant decrease in the number of MOLs in *R‐Ras1* ^*−/−*^, *R‐Ras2* ^*−/−*^ and *R‐Ras1* ^*−/−*^; *R‐Ras2* ^*−/−*^ mice relative to control (***p < .001). (b) Flow cytometry plots representative of total MOL1 population (Mog^+^Egr2^+^) and bar graph showing MOL1 percentage in single and mutant adult mice relative to control. There was a significant increase in the number of MOL1 in *R‐Ras1* ^*−/−*^ (**p < .01), *R‐Ras2* ^*−/−*^ (***p < .001) and *R‐Ras1* ^*−/−*^;*R‐Ras2* ^*−/−*^ (***p < .001) mice. (c) Flow cytometry plots representative of total MOL2 population (Mog^+^Klk6^+^) and bar graph showing MOL2 percentage in single and mutant adult mice relative to control. There was a significant decrease in the number of MOL2 in *R‐Ras1* ^*−/−*^ (**p < .01), *R‐Ras2* ^*−/−*^ (***p < .001) and *R‐Ras1* ^*−/−*^;*R‐Ras2* ^*−/−*^ (***p < .001) adult mice relative to control. (d) Flow cytometry plots representative of total MOL5/6 population (Mog^+^Ptgds^+^) and bar graph showing MOL5/6 percentage in single and mutant adult mice relative to control. There was a significant decrease in the number of MOL5/6 in *R‐Ras1* ^*−/−*^ (**p < .01), *R‐Ras2* ^*−/−*^ (***p < .001) and *R‐Ras1* ^*−/−*^;*R‐Ras2* ^*−/−*^ (***p < .001) adult mice. Bar graphs represents the mean ± SD relative to control. Two‐tailed Student's *t*‐test was used for statistical analysis. SD, standard deviation; *n* = 3 animals per genotype.

Next, we conducted flow cytometry analysis of brain‐derived and Percoll‐enriched oligodendrocyte populations to investigate how absence of R‐Ras1 and/or R‐Ras2 impacts on MOL populations. Our analysis demonstrated that, relative to control, the percentage of Mog^+^ cells in *R‐Ras1KO* mice was ~81% (*p* < .001), in *R‐Ras2KO* ~ 75% (*p* < .001), and in *DKO* mice ~64% (*p* < .001) (Figure 4a). We found that the absence of R‐Ras1 and/or R‐Ras2 led to a profound increase in the MOL1 subpopulation, concomitant with a significant decrease in the MOL2 and MOL5/6 subpopulations (Figure 4b–d). Specifically, the Mog^+^Egr2 MOL1 cell population was increased by close to five‐fold in *R‐Ras1KO* mice (464 ± 214%, *p* < .01), by six‐fold in *R‐Ras2KO* mice (600 ± 138%, *p* < .001), and by four‐fold in *DKO* mice (382 ± 132%, *p* < .001) (Figure 4b). Conversely, the Mog^+^Klk6^+^ MOL2 cell population was decreased in *R‐Ras1KO* mice (72.5 ± 8%, *p* < .01), in *R‐Ras2KO* mice (66 ± 4%, *p* < .001), and in *DKO* mice (63 ± 3.4%, *p* < .001) (Figure 4c). Similarly, the Mog^+^ Ptdgs^+^ MOL5/6 population was reduced in *R‐Ras1KO* mice (80.08 ± 4%, *p* < .01), in *R‐Ras2KO* mice (73 ± 2%, *p* < .001) and in *DKO* mice (71 ± 3%, *p* < .001) (Figure 4d). These data indicate that the absence of R‐Ras1 and/or R‐Ras2 decreases the total number of MOLs (Figure 4a) and alters the proportion of MOL subpopulations in single‐mutant and *DKO* mice (Figure 4b–d).

In addition, we used the optic nerve as another example of a highly myelinated area. In flow cytometry analysis of oligodendrocyte populations enriched by Percoll gradients from optic nerve homogenates, we validated that the MOL1, MOL2, and MOL5/6 subpopulations are also expressed in this tissue (Figure S2a‐c). Analogous to what we found in the whole brain, the absence of R‐Ras1 and/or R‐Ras2 affected the normal distribution of MOL subpopulations in the optic nerve. Specifically, the MOL1 subpopulation increased whereas the MOL2 and MOL5/6 subpopulations decreased in single and double mutant mice (Figure S2a‐c).

**FIGURE 5:**
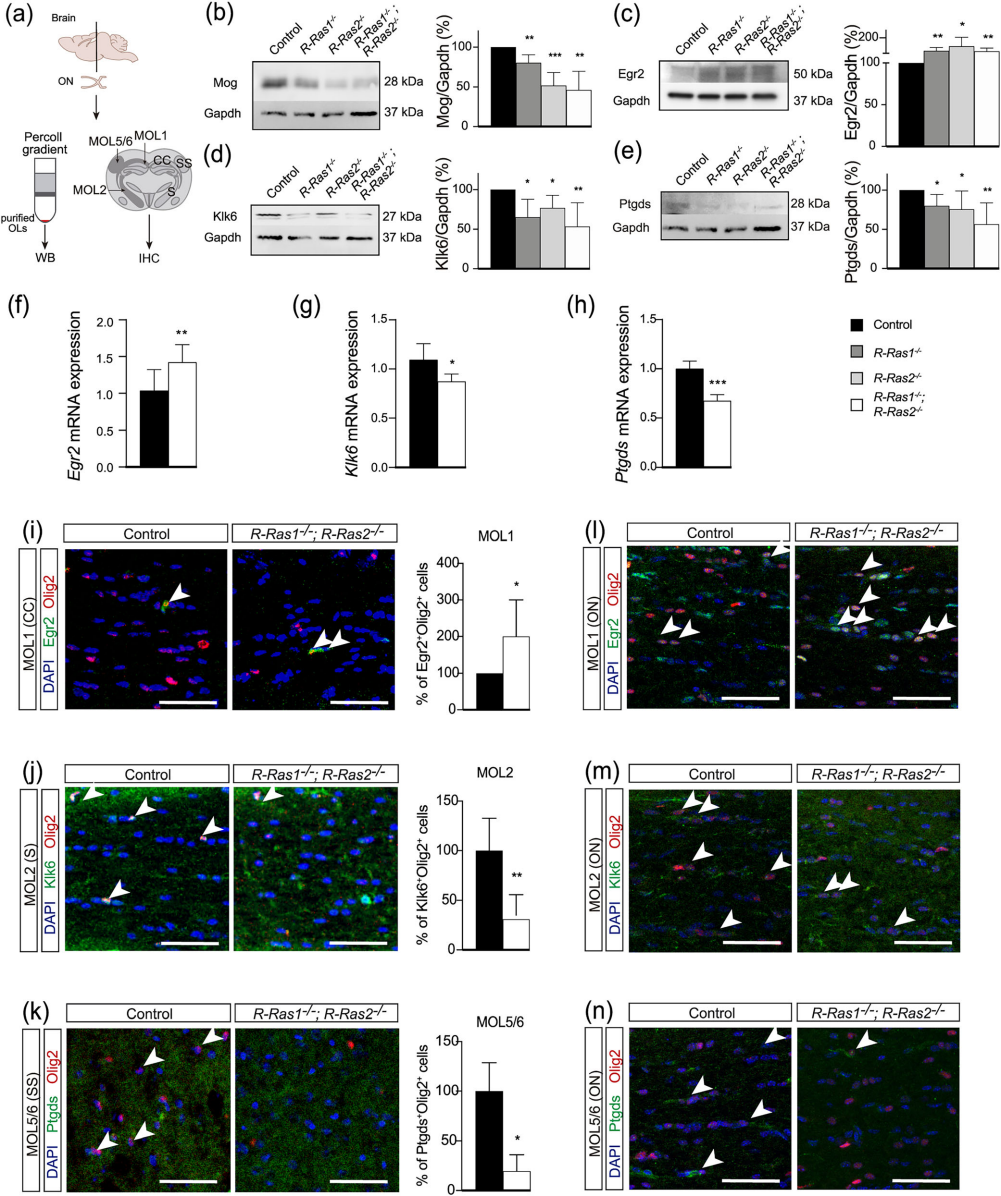
**Western blot and immunohistochemistry confirming R‐Ras1 and/or R‐Ras2 knockout mice have altered relative proportions of MOL1, MOL2, and MOL5/6 subpopulations.** (a) Scheme of the brain area (anteroposterior Bregma −1.7 mm) where all MOL subpopulations are found for subsequent Percoll isolation, western blot analysis and immunohistochemistry. (b) Western blot analysis of Mog (general MOL marker) in oligodendrocyte lysates enriched by Percoll gradients from adult control and mutant mice showed a significant decrease in *R‐Ras1* ^*−/−*^ (**p < .01), *R‐Ras2* ^*−/−*^ (***p < .001) and *R‐Ras1* ^*−/−*^; *R‐Ras2* ^*−/−*^ (**p < .01) mice relative to control. (c) Western blot analysis of Egr2 (MOL1 subpopulation) in optic nerve lysates from adult control and mutant mice showed a significant increase in *R‐Ras1* ^*−/−*^ (**p < .01), *R‐Ras2* ^*−/−*^ (*p < .05) and *R‐Ras1* ^*−/−*^;*R‐Ras2* ^*−/−*^ (**p < .01) mice relative to control. (d) Western blot analysis of Klk6 (MOL2 subpopulation) in oligodendrocyte lysates enriched by Percoll gradients from adult control and mutant mice showed a significant decrease in *R‐Ras1* ^*−/−*^ (*p < .05), *R‐Ras2* ^*−/−*^ (*p < .05) and *R‐Ras1* ^*−/−*^;*R‐Ras2* ^*−/−*^ (**p < .01) mice relative to control. (e) Western blot analysis of Ptgds (MOL5/6 subpopulation) in oligodendrocyte lysates enriched by Percoll gradients from adult control and mutant mice showed a significant decrease in *R‐Ras1* ^*−/−*^ (*p < .05), *R‐Ras2* ^*−/−*^ (*p < .05) and *R‐Ras1* ^*−/−*^;*R‐Ras2* ^*−/−*^ (**p < .01) mice relative to control. (f) RT‐qPCR analysis of *Egr2* in oligodendrocyte lysates from adult control and double mutant mice showed a significant increase in *R‐Ras1* ^*−/−*^;*R‐Ras2* ^*−/−*^ mice (**p < .01) compared to controls. (g) RT‐qPCR analysis of *Klk6* in oligodendrocyte lysates from adult control and double mutant mice demonstrated a significant decrease in *R‐Ras1* ^*−/−*^;*R‐Ras2* ^*−/−*^ mice (*p < .05) compared to controls. (h) RT‐qPCR analysis of *Ptgds* in oligodendrocyte lysates from adult control and double mutant mice revealed a significant decrease in *R‐Ras1* ^*−/−*^;*R‐Ras2* ^*−/−*^ mice (***p < .001) compared to controls. (i) Confocal microscopy images and quantification of MOL1 subpopulation (Egr2^+^Olig2^+^) in corpus callosum (CC) of adult mice showed a significant increase in *R‐Ras1* ^*−/−*^;*R‐Ras2* ^*−/−*^ (*p < .05) mice relative to control. (j) Confocal microscopy images and quantification of MOL2 subpopulation (Klk6^+^Olig2^+^) in striatum (S) of adult mice showed a significant decrease in *R‐Ras1* ^*−/−*^;*R‐Ras2* ^*−/−*^ (**p < .01) mice relative to control. (k) Confocal microscopy images and quantification of MOL5/6 subpopulation (Ptgds^+^Olig2^+^) in somatosensory cortex (SS) of adult mice showed a significant decrease in *R‐Ras1* ^*−/−*^;*R‐Ras2* ^*−/−*^ (*p < .05) mice relative to control. (l) Confocal microscopy images of MOL1 subpopulation (Egr2^+^Olig2^+^) in optic nerve of adult mice showed an increase in *R‐Ras1* ^*−/−*^;*R‐Ras2* ^*−/−*^ mice relative to control. (m) Confocal microscopy images of MOL2 subpopulation (Klk6^+^Olig2^+^) in optic nerve of adult mice showed a decrease in *R‐Ras1* ^*−/−*^;*R‐Ras2* ^*−/−*^ mice relative to control. (n) Confocal microscopy images of MOL5/6 subpopulation (Ptgds^+^Olig2^+^) in optic nerve of adult mice showed a decrease in *R‐Ras1* ^*−/−*^;*R‐Ras2* ^*−/−*^ mice relative to control. Bar graphs represent the mean ± SD relative to wildtype controls. Scale bars, 50 μm. Two‐tailed Student's *t*‐test was used for statistical analysis. SD: Standard deviation; *n* = 4.

We confirmed these results in western blot analysis of Mog expression in Percoll‐enriched oligodendrocytes from brain homogenates (Figure 5a). Mog protein levels were significantly decreased in *R‐Ras1KO* mice (81 ± 10%, *p* < .01), in *R‐Ras2KO* mice (52 ± 17%, *p* < .001), and *DKO* mice (46 ± 24%, *p* < .01) (Figure 5b). For the MOL1 population, Egr2 expression was significantly higher in R‐Ras1, R‐Ras2 and double mutant mice models (Figure 5c). Conversely, for the MOL2 population, Klk6 expression was significantly lower in all mutant mice (Figure 5d) whereas for the MOL5/6 population, Ptgds expression was significantly lower in all our mutant mice (Figure 5e).

At the RNA level, these alterations were confirmed through RT‐qPCR experiments conducted in double mutant mice compared to controls (Figure 5f‐h). In the MOL1 subpopulation, *Egr2* expression was significantly increased in *DKO* mice (1.42 ± 0.24, *p* < .01) compared to controls (1.04 ± 0.28) (Figure 5f). In the MOL2 subpopulation, *Klk6* expression was significantly downregulated in *DKO* mice (0.87 ± 0.08 *p* < .05) compared to controls (1.09 ± 0.16) (Figure 5g). Finally, in the MOL5/6 subpopulation, *Ptgds* expression was also significantly downregulated in *DKO* mice (0.68 ± 0.06, *p* < .001) compared to controls (1.00 ± 0.08) (Figure 5h).

These alterations were further validated with additional markers using flow cytometry, western blot, and RT‐qPCR techniques. The additional markers included FosB for MOL1 identification, Rab37 and Cdkn1c for MOL2, and ApoE for MOL5/6. Analysis of these markers confirmed the increase in the MOL1 subpopulation and the decrease in the MOL2 and MOL5/6 subpopulations in double mutants compared to controls (Figure S3).

In parallel, we reconfirmed these results by immunostaining brain coronal slices (−1.7 mm anteroposterior from Bregma) and optic nerve from adult mice (P90). We co‐stained for the oligodendrocyte lineage marker Olig2 along with the specific subpopulation markers Egr2 for MOL1, Klk6 for MOL2, and Ptgds for MOL5/6. Subsequently, we quantified cells positive for each pair of markers in brain areas enriched for each subpopulation: corpus callosum for MOL1, striatum for MOL2, and somatosensory cortex for MOL5/6. In *DKO* mice, the percentage of Egr2^+^ MOL1 cells was significantly increased relative to control mice (200 ± 100%; *p* < .05) (Figure 5f), whereas we observed significantly decreased percentage of Klk6^+^ MOL2 cells (30.94 ± 20.34%; *p* < .01) and Ptgds^+^ MOL 5/6 cells (19.5 ± 16.53%; *p* < .05) (Figure 5g,h).

We also confirmed these results in the optic nerve. Specifically, in the absence of R‐Ras1 and R‐Ras2, we detected an increase in the number of Egr2^+^ MOL1 cells (Figure 5i) and a decrease in the number of Klk6^+^ MOL2 and Ptgds^+^ MOL5/6 cells relative to control (Figure 5j,k).

Together, these results confirm that in the absence of R‐Ras1 and/or R‐Ras2, the MOL1 subpopulation expands whereas the MOL2 and MOL5/6 subpopulations shrink.

### 3.5 The in vitro overexpression of R‐Ras1 and R‐Ras2 confirms the alterations observed in the MOL1, MOL2, and MOL5/6 subpopulations

To confirm the role that R‐Ras1 and R‐Ras2 play in the specification of different types of MOLs, complementary in vitro gain‐of‐function experiments were conducted. Primary oligodendrocyte cultures were transfected with R‐Ras1 and R‐Ras2, and changes in the proportions of MOLs were evaluated using RT‐qPCR techniques.

**FIGURE 6:**
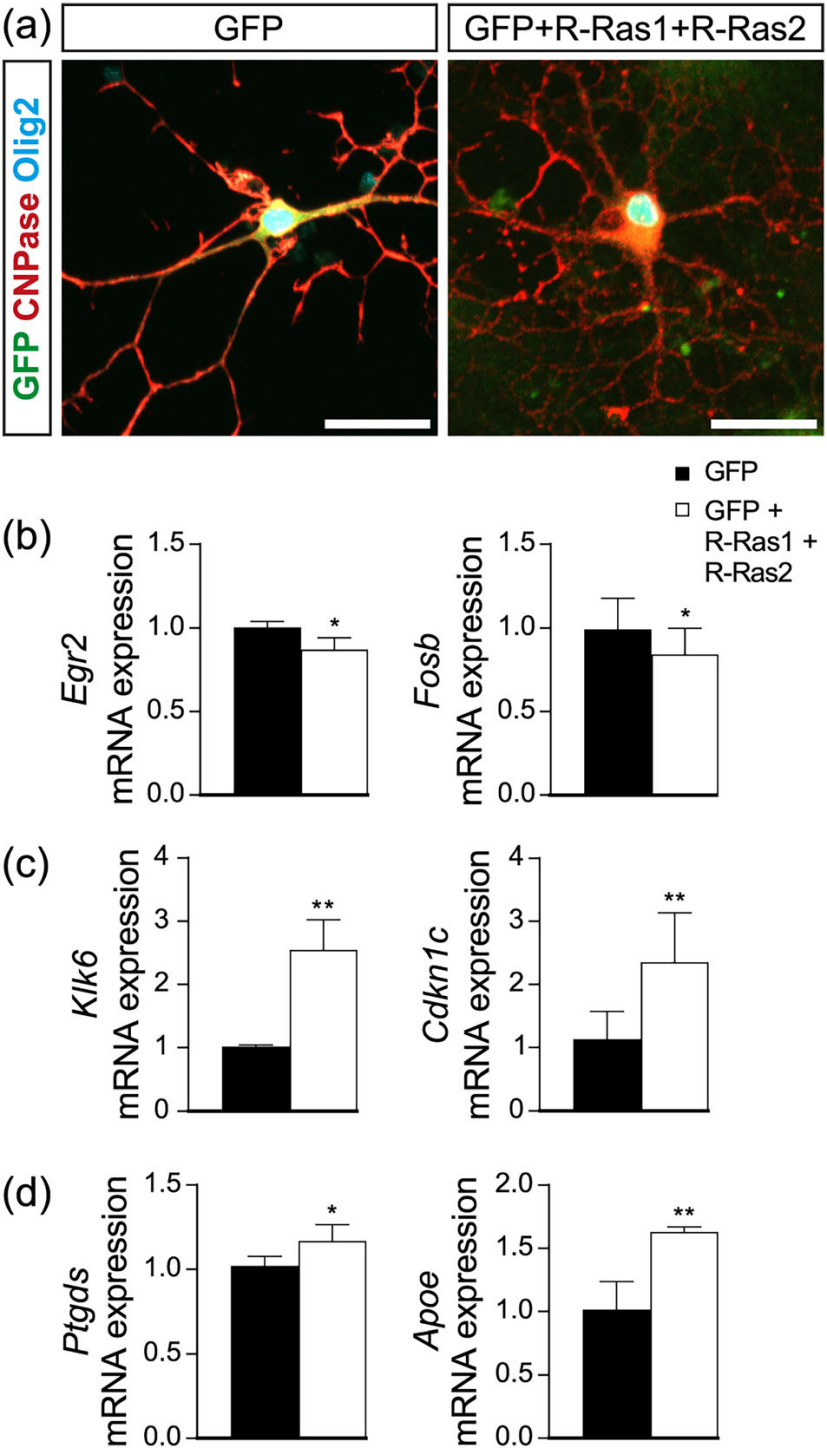
**R‐Ras1 and R‐Ras2 overexpression promotes morphological maturation and alters the distribution of the MOL1, MOL2, and MOL5/6 subpopulations.** (a) Confocal microscopy images of primary oligodendrocyte cultures showing a significant increase in oligodendrocytes with complex morphologies following transfection with GFP + R‐Ras1 + R‐Ras2 (right) compared to control transfection with GFP (left). (b) RT‐qPCR analysis of primary oligodendrocyte cultures displaying a significant decrease in *Egr2* (*p < .05) and *Fosb* (*p < .05) upon transfection with GFP + R‐Ras1 + R‐Ras2 relative to GFP‐transfected cells. (c) RT‐qPCR analysis of primary oligodendrocyte cultures indicating a significant increase in *Klk6* (**p < .01) and *Cdkn1c* (**p < .01) upon transfection with GFPR‐Ras1 + R‐Ras2 relative to GFP‐transfected cells. (d) RT‐qPCR analysis of primary oligodendrocyte cultures revealing a significant increase in *Ptgds* (*p < .05) and *Apoe* (**p < .01) upon transfection with GFP + R‐Ras1 + R‐Ras2 relative to GFP‐transfected cells. Bar graphs represent the mean ± SD relative to wildtype controls. Scale bars, 25 μm. Statistical analysis was performed using a two‐tailed Student's *t*‐test. SD: Standard deviation; *n* = 3 per condition.

The results indicated that overexpression of R‐Ras1 and R‐Ras2 led to a significant decrease in the mRNA expression of *Egr2* (0.87 ± 0.07, *p* < .05) compared to controls transfected with GFP (1.01 ± 0.03). Similarly, *Fosb* mRNA levels were lower (0.84 ± 0.16, *p* < .05) in the R‐Ras1 and R‐Ras2 overexpressing group compared to the GFP controls (0.99 ± 0.18). Conversely, mRNA expression of *Klk6* showed a significant increase (2.55 ± 0.48, *p* < .01) compared to GFP controls (1.01 ± 0.03). Additionally, the mRNA expression of *Cdkn1c* was higher (2.35 ± 0.78, *p* < .01) in the overexpressing group compared to GFP controls (1.13 ± 0.44). Finally, mRNA expression of *Ptgds* increased (1.168 ± 0.1, *p* < .05) compared to controls (1.02 ± 0.06), and *Apoe* levels were elevated (1.63 ± 0.04, *p* < .01) compared to controls (1.01 ± 0.22) (Figure 6).

These results suggest that R‐Ras1 and R‐Ras2 play crucial roles in maintaining the distinct subpopulations of MOLs.

### 3.6 The absence of R‐Ras1 and/or R‐Ras2 differentially affects myelination of axonal groups, altering nerve impulse transmission

**FIGURE 7:**
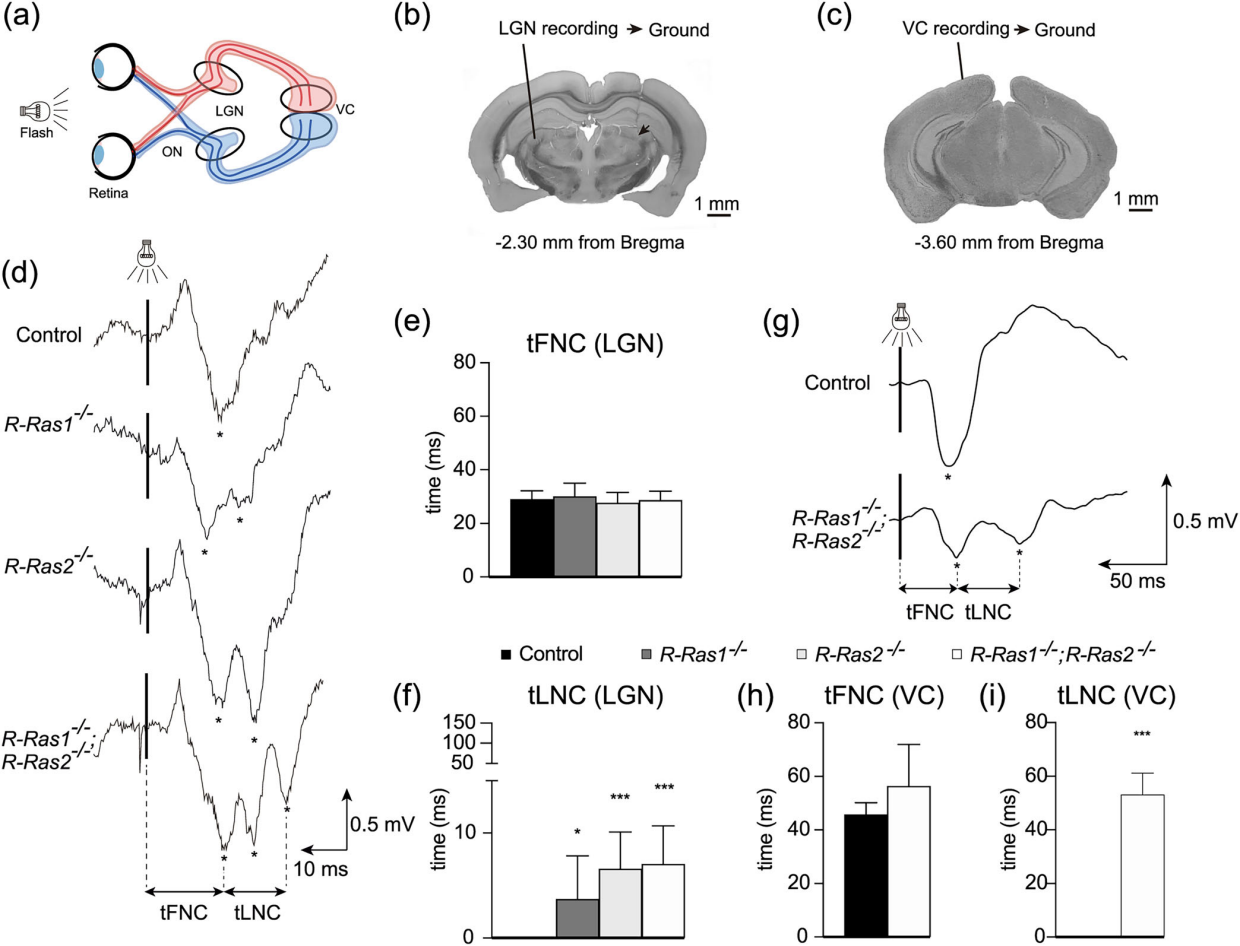
**Visual evoked potential tests suggest presence of axonal groups associated with different degrees of myelination in R‐Ras1 and/or R‐Ras2 knockout mice.** (a) Schematic representation of the visual pathway showing the axonal projections from the retina to the lateral geniculate nucleus (LGN) and the visual cortex (VC). (b) Photomicrograph illustrating the location of implanted electrodes in the LGN. (c) Photomicrograph illustrating the location of the recording electrodes in the VC. (d) Evoked potentials recorded in the LGN from adult (P120) control, *R‐Ras1* ^−/−^, *R‐Ras2* ^−/−^ and *R‐Ras1* ^−/−^;*R‐Ras2* ^−/−^ mice presenting trace deflections (asterisks). Black bar represents the moment of photic stimulation (flash). The average of 20 recordings from representative mice is shown. (e) The First Negative Component (tFNC) in LGN showed no differences on the latency values in mutant mice relative to controls. (f) Latency values from tFNC to the Late Negative Component (tLNC) indicated a significant difference in *R‐Ras1* ^*−/−*^ (*p < .05), *R‐Ras2* ^*−/−*^ (***p < .001) and *R‐Ras1* ^*−/−*^;*R‐Ras2* ^*−/−*^ (***p < .001) adult mice relative to controls. (g) Evoked potentials were recorded from the VC from adult (P120) control and *R‐Ras1* ^−/−^;*R‐Ras2* ^−/−^ mice presenting negative deflections (asterisks). Black bar represents the moment of photic stimulation (flash). The average of 60 recordings from representative control and *R‐Ras1* ^*−/−*^;*R‐Ras2* ^*−/−*^ mice is shown. (h) tFNC in VC showed no differences on the latency values in mutant mice relative to controls. (i) Latency values from tFNC to tLNC indicated a significant difference in *R‐Ras1* ^*−/−*^;*R‐Ras2* ^*−/−*^ (***p < .001) adult mice relative to controls. Bar graphs represent the mean ± SD. Two‐tailed Student's *t*‐test was used for statistical analysis. Scale bar, (b) and (c) 1 mm; (d) 10 ms | 0.5 mV; (g) 50 ms | 0.5 mV. SD, standard deviation; tFNC, time to first negative component; tLNC, time to late negative component.

To assess whether the imbalances in MOL subpopulations observed in the *R‐Ras1KO*, *R‐Ras2KO*, and *DKO* mice impacted on the speed of nerve impulse conduction by specific axonal groups, we performed VEP studies. To this end, we implanted recording electrodes into the dorsal part of the LGN or a stainless‐steel screw in the VC of the mice (Figure 7a). We then performed unilateral stimulation with light flashes to measure the conduction velocity of nerve impulses along the optic nerve in vivo (Figure 7b, arrow). As described previously (Meeren et al., 1998; Wiggins et al., 1982), flash stimulation evokes an early positive–negative–positive field potential, followed by later oscillatory components.

The first negative component of the evoked field potential recorded from the LGN presented with similar times in single‐mutant and *DKO* mice relative to control mice. These latencies were in the range of reported unitary activation of retinal ganglion cell by photic stimulation in mice (Lintas et al., 2013). Specifically, from stimulus to the tFNC (Figure 7d, first asterisk) we did not identify any differences in the latencies among the four groups of mice (control: 29.06 ± 3.14 ms; *R‐Ras1KO*: 30.09 ± 5.01 ms; *R‐Ras2KO*: 27.67 ± 3.94 ms; *DKO*: 27.67 ± 3.35 ms). These data confirm that correctly myelinated axons are present in both single‐mutant and *DKO* mice, suggesting that myelination carried out by at least some MOLs is independent of R‐Ras1 and/or R‐Ras2 (Figure 7d,e).

When we further analyzed the negative component of the evoked LGN field potential, we identified successive drops and a longer total duration in mutant mice compared to controls (Figure 7d, asterisks). Indeed, quantitative analysis of VEPs from the early negative component to the tLNC indicated a significant increase in latency in the *R‐Ras1KO* mice (3.71 ± 4.11 ms, *p* < .05), R‐Ras2KO mice (6.58 ± 3.52 ms, *p* < .001), and *DKO* mice (6.58 ± 3.63 ms, *p* < .001) compared to the controls (Figure  7d,f).

Furthermore, we measured the total conduction velocities of the whole visual pathway from retina to VC (Figure 7c). Consistent to what was found in the LGN, from stimulus to tFNC we did not identify any differences in the latencies between *DKO* (56.33 ± 15.54 ms) and control mice (45.67 ± 4.51 ms) (Figure 7g, first asterisk). Again, these data confirmed the presence of correctly myelinated axons along the visual pathway in *DKO* mice (Figure 7g,h). However, we did identify successive drops and a longer latency in mutant mice compared to controls. Specifically, the latency from tFNC to tLNC indicated a significant increase in latency in the *DKO* mice (53.25 ± 7.89 ms, *p* < .001) compared to controls (Figure 7g,i).

The successive negative waves registered in both the LGN, and the VC recordings indicated a decrease in the conduction speed of axons, consistent with the existence of axonal groups with different degrees of myelination.

The first depolarization wave found on the VEPs analyses did not show modifications in mutant mice relative to control. This finding is consistent with the existence of a population of wide axons whose myelination is non‐dependent of R‐Ras1 and/or R‐Ras2. However, more successive negative waves were found on the mutant mice, compatible with the presence of unmyelinated thin axons in the absence of these GTPases (Figure 7).

## 4 DISCUSSION

In previous studies, we reported that R‐Ras1 and R‐Ras2 are expressed in oligodendrocytes and participate in CNS myelination (Alcover‐Sanchez et al., 2021; Sanz‐Rodriguez et al., 2018). However, deeper investigation revealed variable impacts on myelination of optic nerve axons in the absence of R‐Ras1 and/or R‐Ras2. Specifically, the presence of correctly myelinated axons in mutant mice suggest that loss of R‐Ras1 and/or R‐Ras2 does not affect all oligodendrocyte populations involved in myelination in the same way. Based on our findings, we hypothesize that these GTPases have distinct roles in regulating different MOL subpopulations.

Our classification of oligodendrocytes in the optic nerve based on the caliber of the axons they myelinate provided us with valuable insights. Our results indicated that R‐Ras1 and R‐Ras2 are specifically important for ensuring the myelination of thinner axons. Specifically, we found that absence of R‐Ras1 was associated with a reduction in the thickness of the myelin sheath (resulting from fewer layers wrapping around the axon) without affecting the number of myelinated axons. Conversely, the absence of R‐Ras2 was associated with a reduced percentage of thin myelinated axons. Consequently, mice lacking both R‐Ras1 and R‐Ras2 had an even more pronounced decrease in the percentage of thin myelinated axons. In contrast, lack of R‐Ras GTPases did not have a significant impact on the myelination of wide axons, suggesting a specific and additive role for R‐Ras1 and R‐Ras2 in the oligodendrocytes responsible for myelination of thin axons, independent of myelination of thicker axons.

However, other factors could influence the myelination of wide axons, such as the intrinsic preference of oligodendrocytes to myelinate larger caliber axons. In addition, it is possible that neuronal R‐Ras1 and/or R‐Ras2 expression itself could contribute to the myelination processes. Nevertheless, it is important to highlight that previous in vivo work from our laboratory demonstrated that R‐Ras1 and R‐Ras2 play an intrinsic role in the oligodendrocyte lineage, independent of the interaction with the axons that they myelinate. In this sense, in vitro studies of R‐Ras1‐ and R‐Ras2‐deficient oligodendrocytes revealed altered survival and differentiation even in the absence of neurons (Alcover‐Sanchez et al., 2021; Garcia‐Martin et al., 2022; Sanz‐Rodriguez et al., 2018). These results strongly support the idea of intrinsic functions for R‐Ras1 and R‐Ras2 in oligodendrocytes, independent of their interaction with neurons.

However, to substantiate this claim, the use of conditional knockout mice for R‐Ras1, R‐Ras2, and R‐Ras1/R‐Ras2 is required. This would enable a more precise evaluation of the specific functions that these GTPases perform in oligodendrocytes while minimizing potential interference from other cell types. These aspects will be addressed in future research.

In flow cytometry experiments, we revealed a heterogeneous distribution of R‐Ras1 and/or R‐Ras2 expression in the overall MOL population, which may explain why the lack of one or both GTPases specifically altered the proportion of MOL subpopulations, and impacted on the myelination of axonal groups. The presence of correctly myelinated axons in the mutant mice supports the existence of some remaining MOL population, independent of R‐Ras1 and/or R‐Ras2, that seems to not be affected by deficiency in these GTPases.

The in vivo VEP analysis of single‐mutant and *DKO* mice suggested the presence of axonal groups with different degrees of myelination, in line with the different degrees of hypomyelination observed in the absence of R‐Ras1 and/or R‐Ras2 (Alcover‐Sanchez et al., 2021; Sanz‐Rodriguez et al., 2018). However, it is important to highlight that no alterations were identified from the stimulus to the tFNC, which supports the presence of correctly myelinated wide axons in both single‐mutant and *DKO* mice. These results confirm a role for R‐Ras1 and R‐Ras2 in the diversity of oligodendrocytes and that a MOL subpopulation that is independent of R‐Ras1 and/or R‐Ras2 carries out some level of myelination. At a broader level, our work aligns well with previous findings about MOL heterogeneity (https://brainrnaseq.org/, http://linnarssonlab.org/oligodendrocytes/) (Marques et al., 2016; Pérez‐Cerdá et al., 2015; Simons & Nave, 2016).

To date, six molecularly distinct MOL subpopulations have been identified in different regions and physiological states within the CNS, each characterized by unique gene expression profiles (Falcão et al., 2018; Floriddia et al., 2020; Hilscher et al., 2022; Jäkel et al., 2019; Marques et al., 2016). In this study, we chose to analyze MOL1, MOL2, and MOL5/6 as the most genetically different subpopulations. MOL2 and MOL5/6 are the most abundant subpopulations in the adult CNS (Marques et al., 2016). In contrast, RNAscope and in situ sequencing techniques have suggested that MOL1 is a transient injury‐associated state present in multiple sclerosis disease and in spinal cord injury models (Falcão et al., 2018; Floriddia et al., 2020; Hilscher et al., 2022).

The modest differences in the expression of R‐Ras1 and/or R‐Ras2 among the MOL1, MOL2, and MOL5/6 subpopulations do not imply that these GTPases are exclusive to these subpopulations. Instead, they suggest an alternative classification based on the R‐Ras1 and/or R‐Ras2 expression levels. However, various methods—including flow cytometry, RT‐qPCR, western blot, immunohistochemistry, and in vitro overexpression experiments—demonstrated that alterations in the R‐Ras1 and/or R‐Ras2 expression levels significantly affected the relative proportions of the analyzed MOL subpopulations.

Thus, the absence of R‐Ras1 and/or R‐Ras2 would negatively affect the differentiation of the MOL2 and MOL5/6 subpopulations while providing a competitive advantage to the MOL1 subpopulation, suggesting that these GTPases play a central role in generating the heterogeneity of MOLs due to specific signaling mediated by R‐Ras1 and/or R‐Ras2. On the other hand, the increase in MOL1 could also be due to a state of injury or a general blockade in differentiation. In this sense, the altered myelination state of the single‐mutant and *DKO* mice might be recognized as damage, thus inducing MOL1 proliferation as a response to the myelin deficiency.

The observed increase in the MOL1 subpopulation in the absence of R‐Ras1 and/or R‐Ras2 is consistent with our previous research, in which we described how mutant mice deficient in R‐Ras1 and R‐Ras2 presented a decrease in oligodendrocyte populations, associated with a myelin deficiency and axonal degeneration (Alcover‐Sanchez et al., 2021).

Given that the gene expression profile of MOL1 is associated with processes of cell mobility and adhesion (Marques et al., 2016), we hypothesize that the MOL1 subpopulation may represent a precursor stage in the maturation process of MOLs toward MOL5/6. Therefore, the observed increase in the MOL1 subpopulation in the R‐Ras1 and R‐Ras2 mutants could indicate a blockage in the differentiation process of MOLs. Previous studies have reported that as myelination progresses, the MOL1 subpopulation is gradually replaced by MOL5/6, ultimately constituting a minority percentage of the total MOLs in the adult CNS (Marques et al., 2016). It is important to highlight that these potential roles are inferred from gene expression patterns and require further validation through additional functional studies.

The alterations to the proportion of MOL subpopulations observed in mutant mice lacking R‐Ras1 and/or R‐Ras2 support the idea that these GTPases participate in the proper establishment of oligodendrocyte heterogeneity. Moreover, the overexpression experiments of R‐Ras1 and R‐Ras2 provide further support for the hypothesis that these GTPases play a critical role in establishing the proportions of the MOL subpopulations.

In this sense, it is important to highlight that extrinsic signals have been proposed to be responsible for the heterogeneity of MOLs (Bechler et al., 2015; Tomassy et al., 2014; Tripathi et al., 2019; Zonouzi et al., 2019). Given that the R‐Ras1 and R‐Ras2 GTPases are activated by a wide variety of extracellular stimuli, they could be a key element in the regulation of the molecular mechanisms involved in the survival, differentiation, and specification of MOLs.

In summary, the functional diversity of oligodendrocytes and their ability to adapt to changing environments are components of homeostasis and proper functioning of the CNS. Therefore, uncovering molecular mechanisms responsible for this heterogeneity, such as those regulated by R‐Ras1 and R‐Ras2, is crucial for understanding the myelination processes and for identifying strategies for regeneration and repair of the nervous system. In this way, the importance of R‐Ras1 and R‐Ras2 in the regulation of oligodendrocyte heterogeneity contributes to a deeper understanding of the mechanisms underlying the functional diversity of these cells and their relevance in diseases of the CNS.

## AUTHOR CONTRIBUTIONS

Conceptualization, B.C.; methodology, B.A.‐S., G.G.‐M., V.P.‐G., and B.C.; validation, B.C.; formal analysis, B.A.‐S., G.G.‐M. and B.C.; writing–original draft preparation B.C.; supervision, F.W., A.D. A.Q., A.G., JM.D.‐G., M.P.P., P.V. and B.C. All authors have read and agreed to the published version of the manuscript.

## CONFLICT OF INTEREST STATEMENT

The authors of the article titled “R‐Ras1 and R‐Ras2 regulate mature oligodendrocyte subpopulations” submitted for consideration in GLIA. Hereby, we declare that there is no conflict of interest that could influence our objectivity in the research or presentation of the results of this work.

We confirm that we have no financial interests or relationships with any organization that may have a direct or indirect financial interest in the subject matter discussed in this article. Furthermore, we have no personal, professional, or academic conflicts of interest that could affect the impartiality of our research or the interpretation of the results.

## Supporting information


**Supplementary Figure 1: Gating strategy for MOL1, MOL2, and MOL5/6 subpopulations.** (a) Egr2 gating strategy illustrated with representative dot plots comparing negative control versus Egr2^+^ staining, along with a histogram of Egr2 controls. (b) Klk6 gating strategy depicted with representative dot plots comparing negative control versus Klk6^+^ staining, accompanied by a histogram of Klk6 controls. (c) Ptgds gating strategy presented with representative dot plots comparing negative control versus Ptgds^+^ staining, along with a histogram of Ptgds controls. (a–c) Histograms represent the negative control in black and the positive control in red. Asterisks indicate the positive threshold for each marker.

**Supplementary Figure 2. Relative proportion of MOL1, MOL2 and MOL5/6 is altered in the absence of R‐Ras1 and/or R‐Ras2 in the ON.** (a) Flow cytometry plots representative of total MOL1 population (Mog^+^Egr2^+^) and bar graph showing MOL1 percentage in single and mutant adult mice relative to control. There was a significant increase in the number of MOL1 in *R‐Ras1* ^*−/−*^ (***p < .001), *R‐Ras2* ^*−/−*^ (*p < .05) and *R‐Ras1* ^*−/−*^;*R‐Ras2* ^*−/−*^ (***p < .001). (b) Flow cytometry plots representative of total MOL2 population (Mog^+^Klk6^+^) and bar graph showing MOL2 percentage in single and mutant adult mice relative to control. There was a significant decrease in the number of MOL2 in *R‐Ras1* ^*−/−*^ (*p < .05), *R‐Ras2* ^*−/−*^ (*p < .05) and *R‐Ras1* ^*−/−*^;*R‐Ras2* ^*−/−*^ (**p < .01) adult mice relative to control. (c) Flow cytometry plots representative of total MOL5/6 population (Mog^+^Ptgds^+^) and bar graph showing MOL5/6 percentage in single and mutant adult mice relative to control. There was a significant decrease in the number of MOL5/6 in *R‐Ras1* ^*−/−*^ (***p < .001), *R‐Ras2* ^*−/−*^ (***p < .001) and *R‐Ras1* ^*−/−*^;*R‐Ras2* ^*−/−*^ (***p < .001) adult mice relative to control. Bar graphs represent the mean ± SD relative to control. Two‐tailed Student's *t*‐test was used for statistical analysis. SD, standard deviation. *n* = 3 animals per genotype.

**Supplementary Figure 3. Confirmation of altered relative proportions of MOL1, MOL2, and MOL5/6 subpopulations in R‐Ras1 and/or R‐Ras2 knockout mice through RT‐qPCR, flow cytometry and western blot analysis**. (a) Representative histogram and bar graph of FosB^+^ subpopulation geometric mean fluorescence showing an increase in the MOL1 subpopulation in *R‐Ras1* ^*−/−*^;*R‐Ras2* ^*−/−*^ mice (*p < .05) compared to control. (b) Representative histogram and bar graph of Rab37^+^ subpopulation geometric mean fluorescence indicating a decrease in the MOL2 subpopulation in *R‐Ras1* ^*−/−*^;*R‐Ras2* ^*−/−*^ mice (*p < .05) compared to controls. (c) Representative histogram and bar graph of ApoE^+^ subpopulation geometric mean fluorescence revealing a decrease in the MOL5/6 subpopulation in *R‐Ras1* ^*−/−*^;*R‐Ras2* ^*−/−*^ mice (***p < .001) compared to controls. (d) Western blot analysis of FosB (MOL1 subpopulation) in oligodendrocyte lysates enriched by Percoll gradients from adult control and mutant mice, showing a significant increase in *R‐Ras1* ^*−/−*^;*R‐Ras2* ^*−/−*^ mice (**p < .01) compared to controls. (e) Western blot analysis of Rab37 (MOL2 subpopulation) in oligodendrocyte lysates enriched by Percoll gradients from adult control and mutant mice, revealing a significant decrease in *R‐Ras1* ^*−/−*^;*R‐Ras2* ^*−/−*^ mice (*p < .05) compared to controls. (f) Western blot analysis of ApoE (MOL5/6 subpopulation) in oligodendrocyte lysates enriched by Percoll gradients from adult control and mutant mice, illustrating a significant decrease in *R‐Ras1* ^*−/−*^;*R‐Ras2* ^*−/−*^ mice (*p < .05) compared to controls. (g) RT‐qPCR of *Fosb* in optic nerve lysates from adult control and double mutant mice, showing a significant increase in *R‐Ras1* ^*−/−*^;*R‐Ras2* ^*−/−*^ mice (***p < .001) compared to controls. (h) RT‐qPCR of *Cdkn1c* in oligodendrocyte lysates from adult control and double mutant mice, displaying a significant decrease in *R‐Ras1* ^*−/−*^;*R‐Ras2* ^*−/−*^ mice (***p < .001) compared to control. (i) RT‐qPCR of *Apoe* in oligodendrocyte lysates from adult control and double mutant mice, illustrating a significant decrease in *R‐Ras1* ^*−/−*^;*R‐Ras2* ^*−/−*^ mice (**p < .01) compared to control. Bar graphs represent the mean ± SD relative to wildtype controls. Statistical analysis was performed using a two‐tailed Student's *t*‐test. SD: standard deviation; *n* = 3 per genotype.

## Data Availability

The data that support the findings of this study are available from the corresponding author upon reasonable request.
