# Supplementary figures and images for: R‐Ras1 and R‐Ras2 regulate mature oligodendrocyte subpopulations
Source: Glia. 2024 Nov 19;73(4):701–19. doi: 10.1002/glia.24643 (PMC11845848; doi:10.1002/glia.24643)

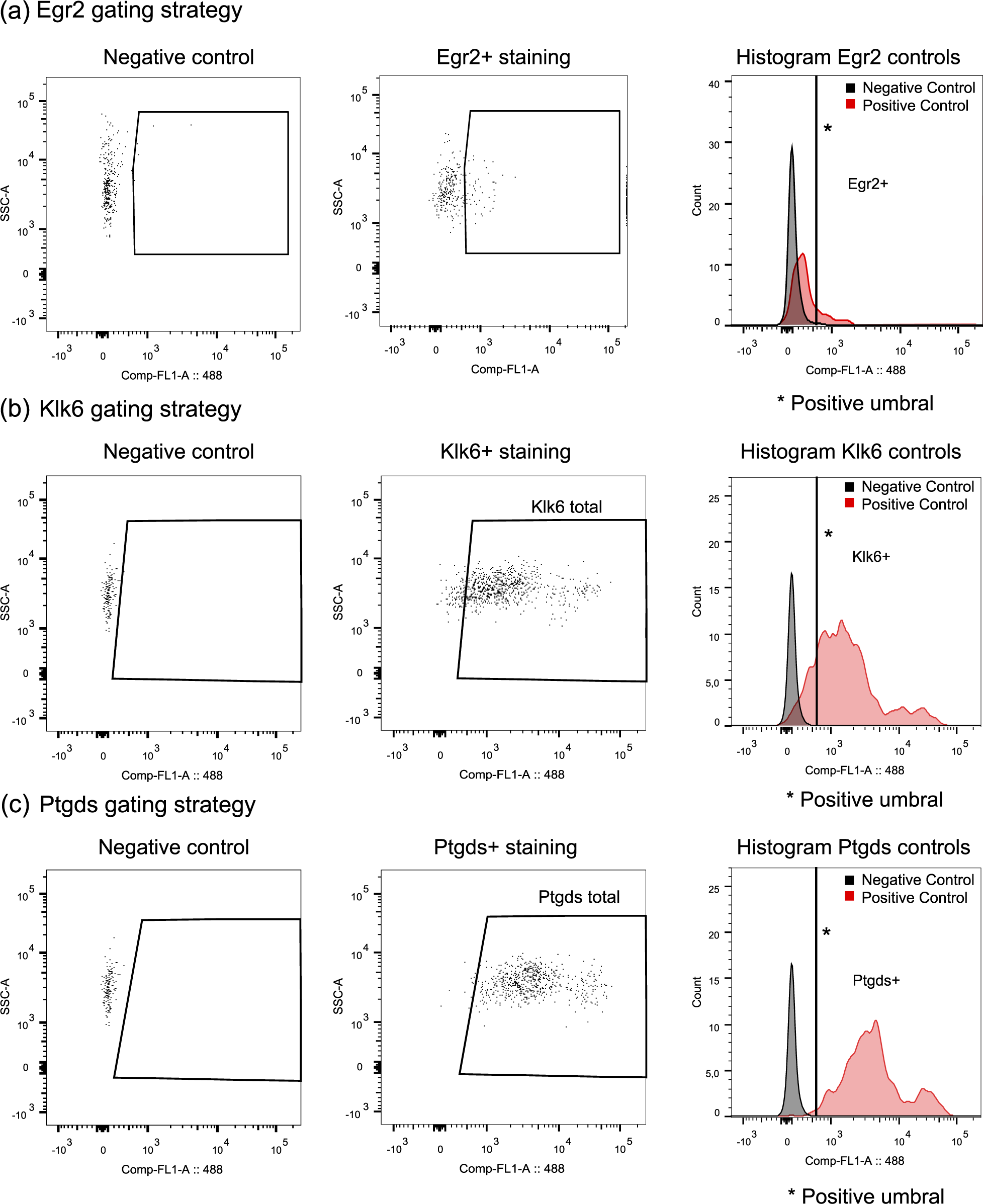

Supplement: Supplementary file 1 — Supplementary Figure 1: Gating strategy for MOL1, MOL2, and MOL5/6 subpopulations. (a) Egr2 gating strategy illustrated with representative dot plots comparing negative control versus Egr2+ staining, along with a histogram of Egr2 controls. (b) Klk6 gating strategy depicted with representative dot plots comparing negative control versus Klk6+ staining, accompanied by a histogram of Klk6 controls. (c) Ptgds gating strategy presented with representative dot plots comparing negative control versus Ptgds+ staining, along with a histogram of Ptgds controls. (a–c) Histograms represent the negative control in black and the positive control in red. Asterisks indicate the positive threshold for each marker. [file GLIA-73-701-s003.tif]

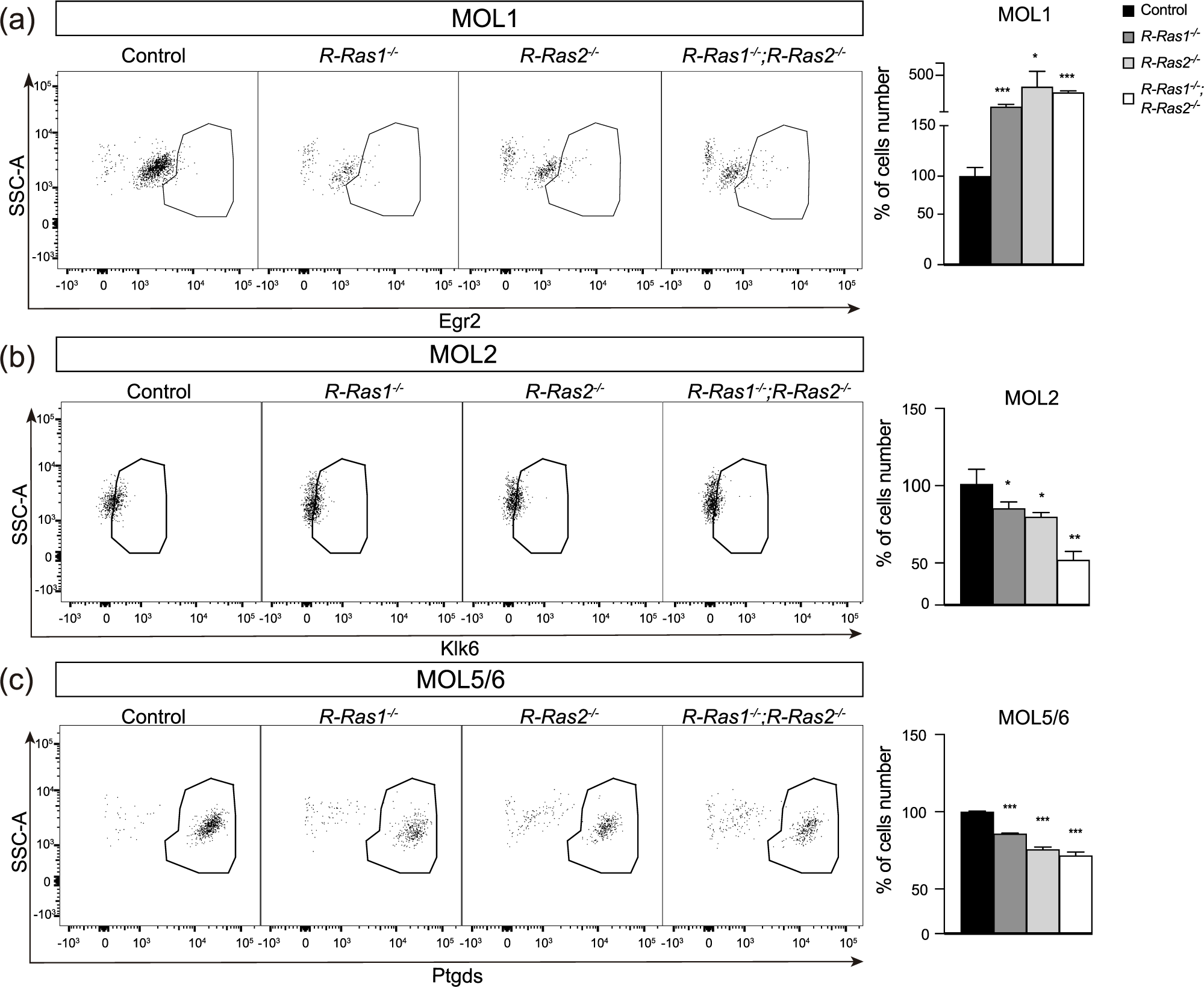

Supplement: Supplementary file 2 — Supplementary Figure 2. Relative proportion of MOL1, MOL2 and MOL5/6 is altered in the absence of R‐Ras1 and/or R‐Ras2 in the ON. (a) Flow cytometry plots representative of total MOL1 population (Mog+Egr2+) and bar graph showing MOL1 percentage in single and mutant adult mice relative to control. There was a significant increase in the number of MOL1 in R‐Ras1 −/− (***p < .001), R‐Ras2 −/− (*p < .05) and R‐Ras1 −/− ;R‐Ras2 −/− (***p < .001). (b) Flow cytometry plots representative of total MOL2 population (Mog+Klk6+) and bar graph showing MOL2 percentage in single and mutant adult mice relative to control. There was a significant decrease in the number of MOL2 in R‐Ras1 −/− (*p < .05), R‐Ras2 −/− (*p < .05) and R‐Ras1 −/− ;R‐Ras2 −/− (**p < .01) adult mice relative to control. (c) Flow cytometry plots representative of total MOL5/6 population (Mog+Ptgds+) and bar graph showing MOL5/6 percentage in single and mutant adult mice relative to control. There was a significant decrease in the number of MOL5/6 in R‐Ras1 −/− (***p < .001), R‐Ras2 −/− (***p < .001) and R‐Ras1 −/− ;R‐Ras2 −/− (***p < .001) adult mice relative to control. Bar graphs represent the mean ± SD relative to control. Two‐tailed Student's t‐test was used for statistical analysis. SD, standard deviation. n = 3 animals per genotype. [file GLIA-73-701-s001.tif]

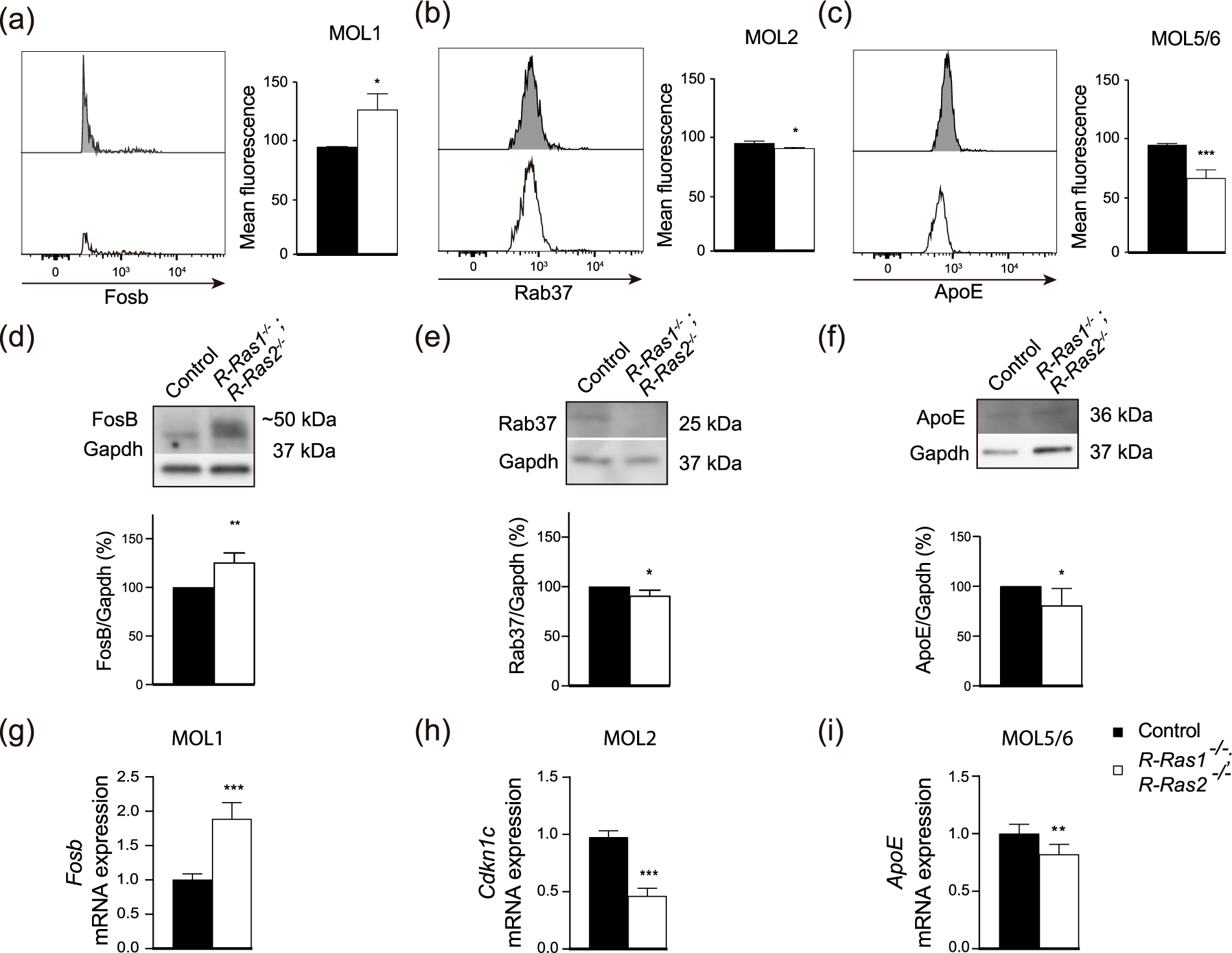

Supplement: Supplementary file 3 — Supplementary Figure 3. Confirmation of altered relative proportions of MOL1, MOL2, and MOL5/6 subpopulations in R‐Ras1 and/or R‐Ras2 knockout mice through RT‐qPCR, flow cytometry and western blot analysis. (a) Representative histogram and bar graph of FosB+ subpopulation geometric mean fluorescence showing an increase in the MOL1 subpopulation in R‐Ras1 −/− ;R‐Ras2 −/− mice (*p < .05) compared to control. (b) Representative histogram and bar graph of Rab37+ subpopulation geometric mean fluorescence indicating a decrease in the MOL2 subpopulation in R‐Ras1 −/− ;R‐Ras2 −/− mice (*p < .05) compared to controls. (c) Representative histogram and bar graph of ApoE+ subpopulation geometric mean fluorescence revealing a decrease in the MOL5/6 subpopulation in R‐Ras1 −/− ;R‐Ras2 −/− mice (***p < .001) compared to controls. (d) Western blot analysis of FosB (MOL1 subpopulation) in oligodendrocyte lysates enriched by Percoll gradients from adult control and mutant mice, showing a significant increase in R‐Ras1 −/− ;R‐Ras2 −/− mice (**p < .01) compared to controls. (e) Western blot analysis of Rab37 (MOL2 subpopulation) in oligodendrocyte lysates enriched by Percoll gradients from adult control and mutant mice, revealing a significant decrease in R‐Ras1 −/− ;R‐Ras2 −/− mice (*p < .05) compared to controls. (f) Western blot analysis of ApoE (MOL5/6 subpopulation) in oligodendrocyte lysates enriched by Percoll gradients from adult control and mutant mice, illustrating a significant decrease in R‐Ras1 −/− ;R‐Ras2 −/− mice (*p < .05) compared to controls. (g) RT‐qPCR of Fosb in optic nerve lysates from adult control and double mutant mice, showing a significant increase in R‐Ras1 −/− ;R‐Ras2 −/− mice (***p < .001) compared to controls. (h) RT‐qPCR of Cdkn1c in oligodendrocyte lysates from adult control and double mutant mice, displaying a significant decrease in R‐Ras1 −/− ;R‐Ras2 −/− mice (***p < .001) compared to control. (i) RT‐qPCR of Apoe in oligodendrocyte lysates from adult [file GLIA-73-701-s002.tif]
